# Supplementary material for: Open Modification Searching of SARS-CoV-2–Human Protein Interaction Data Reveals Novel Viral Modification Sites
Source: Mol Cell Proteomics. 2022 Oct 12;21(12):100425. doi: 10.1016/j.mcpro.2022.100425 (PMC9554009; doi:10.1016/j.mcpro.2022.100425)
Supplement: Supplementary Figures [file mmc1.pdf]

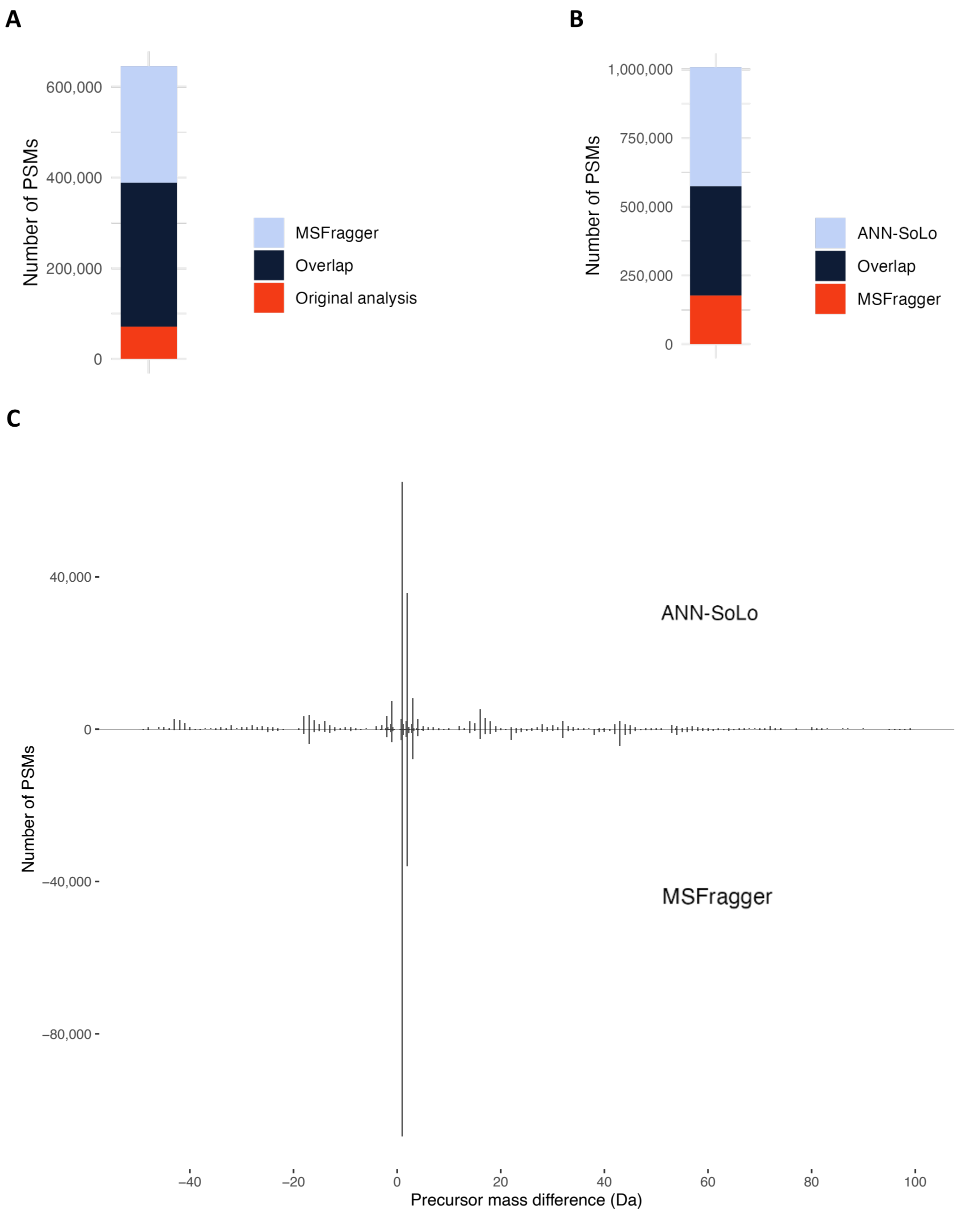

Fig. S1. PSM results from open modification searching. (A) Comparison in identification performance between the originally reported results using standard searching and open modification searching with MSFragger. Open modification searching was able to identify more spectra, corresponding to the identification of a large number of modified PSMs. **(B)** Comparison of the identification results from ANN-SoLo with the identification results of MSFragger. **(C)** Mirror plot illustrating the precursor mass differences observed by open modification searching with ANN-SoLo (top) and MSFragger (bottom).

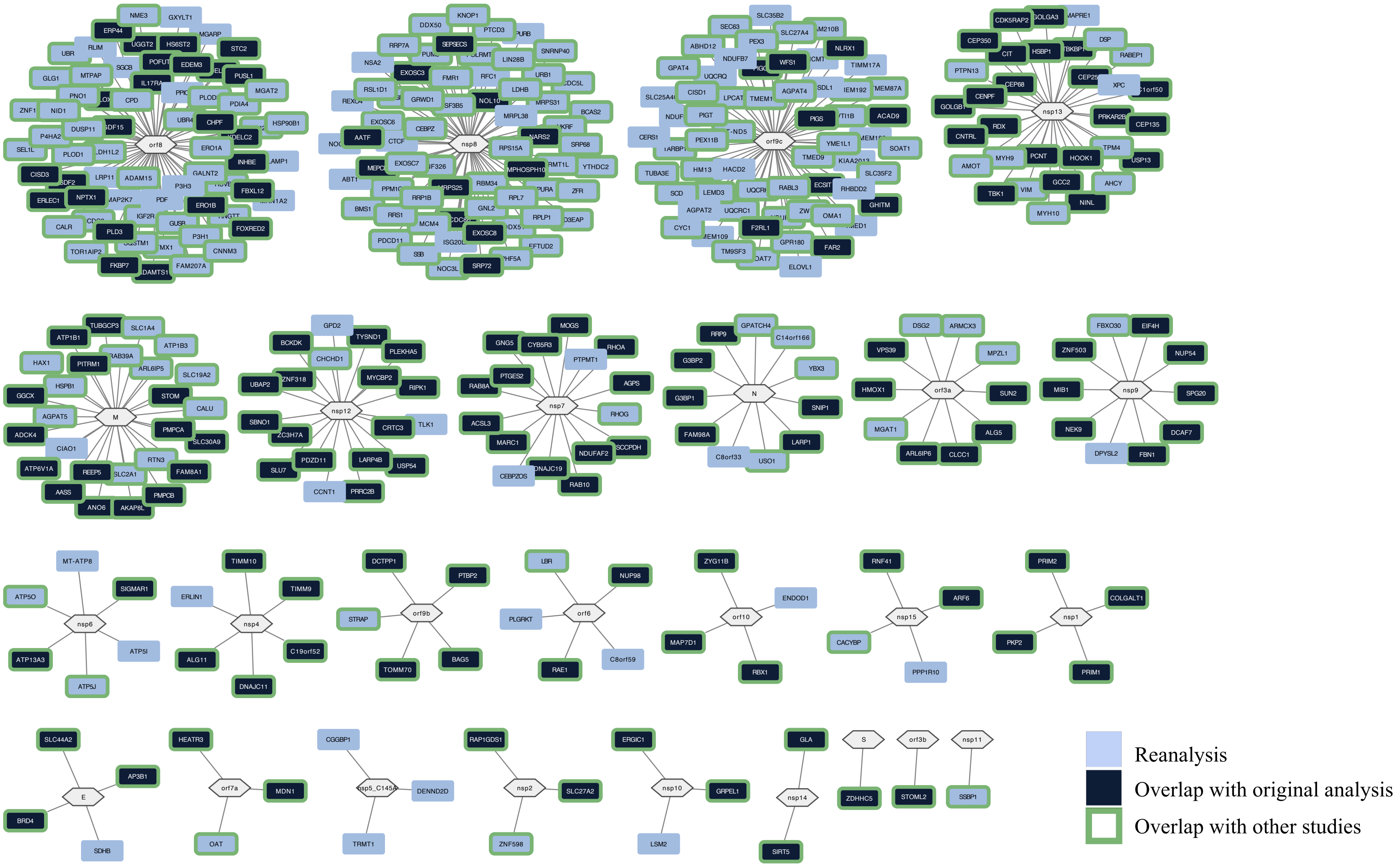

Fig. S2. PPI network. Interaction network of the 375 identified PPIs, including 164 interactions that overlap in the original study, and 124 interactions that were found in independent SARS-CoV-2 interactome studies.

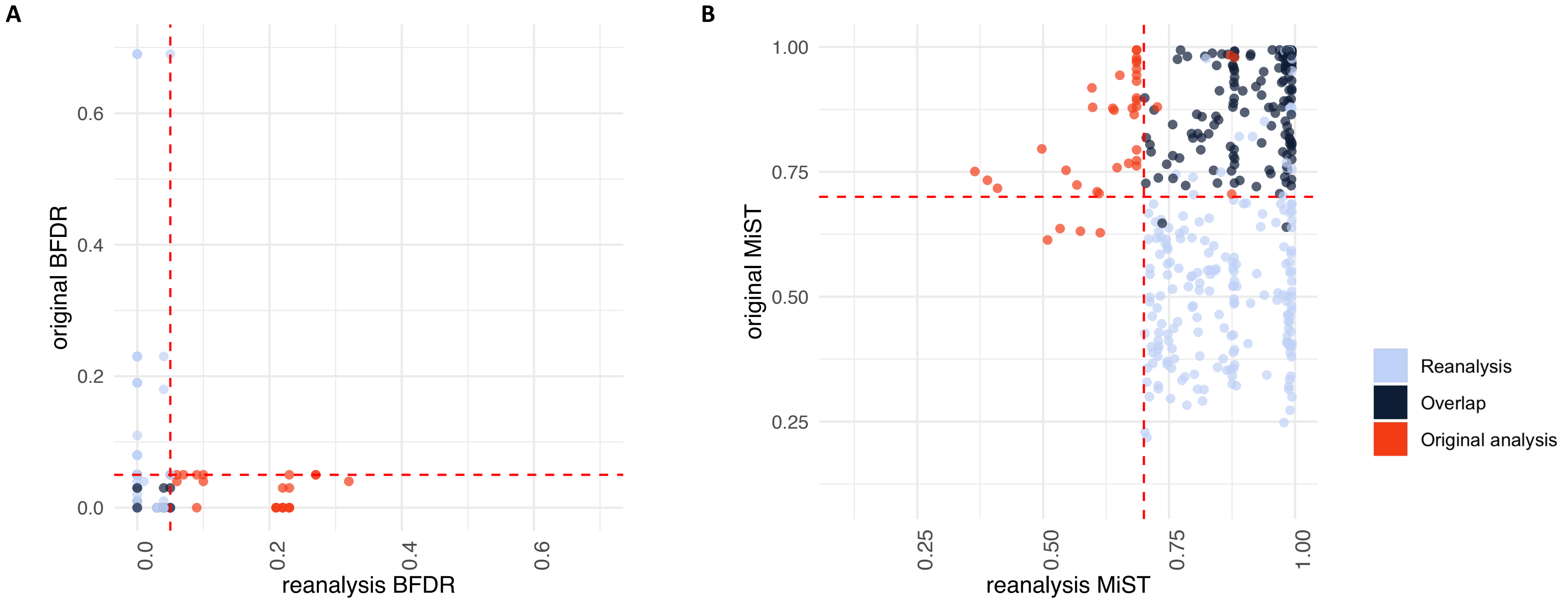

Fig. S3. Evaluation of the PPI filtering thresholds. 363 of the 375 PPIs identified in the reanalysis are present in the unfiltered results of the original analysis. 203 of the 332 identified PPIs in the original analysis are present in the unfiltered results of the reanalysis. Here we show **(A)** the SAINTexpress BFDR and **(B)** the MiST scores of the original analysis plotted against those of the reanalysis for 402 identified PPIs present in both unfiltered results. The red dotted lines indicate the filtering thresholds used for the first step in the original two-step filtering strategy, which is the same as the thresholds used during the reanalysis (SAINTexpress BFDR ≤ 0.05, MiST score ≥ 0.7).

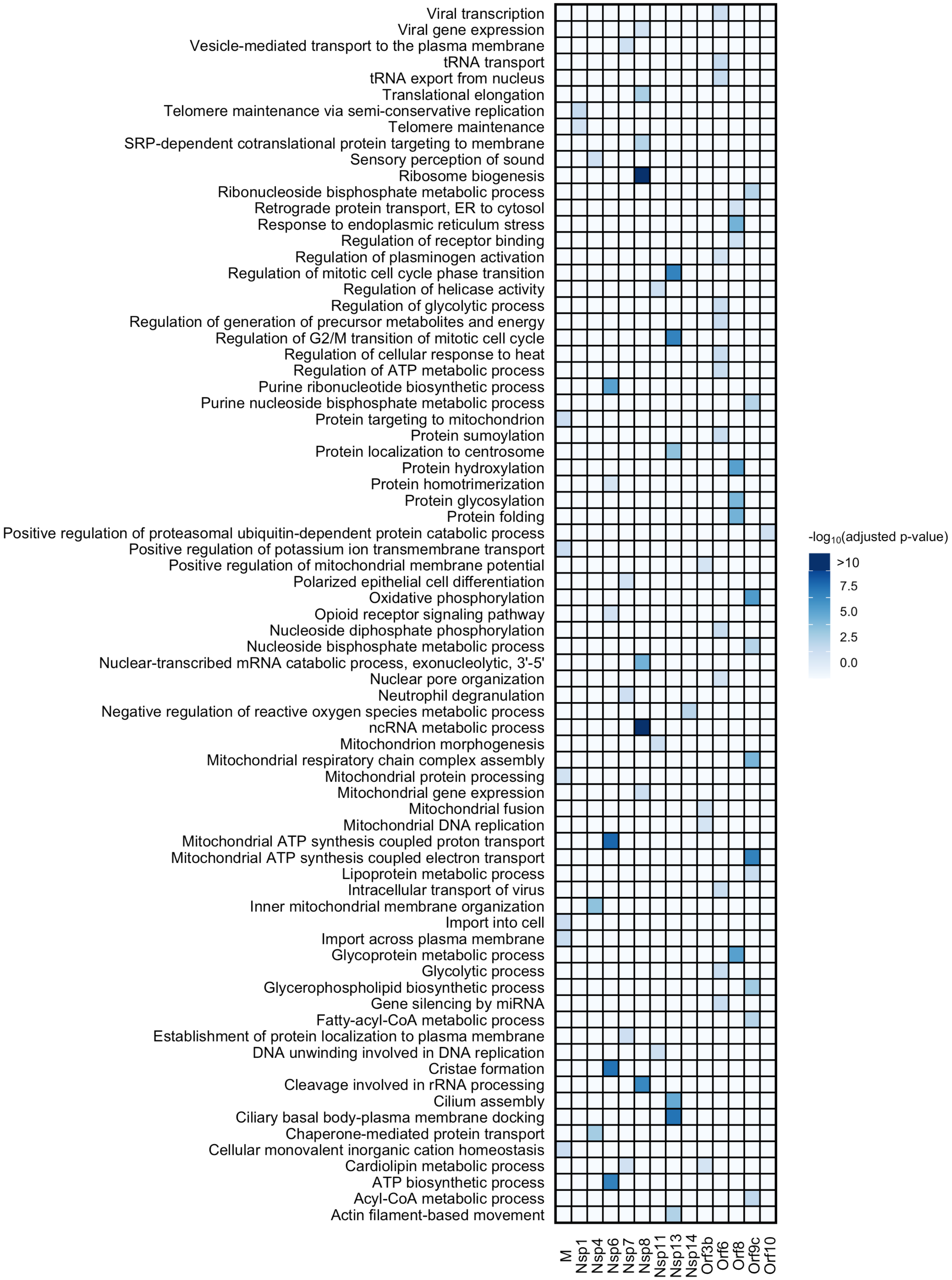

Fig. S4. GO enrichment analysis. Heatmap showing all GO terms from the GO enrichment analysis for each of the SARS-CoV-2 proteins.

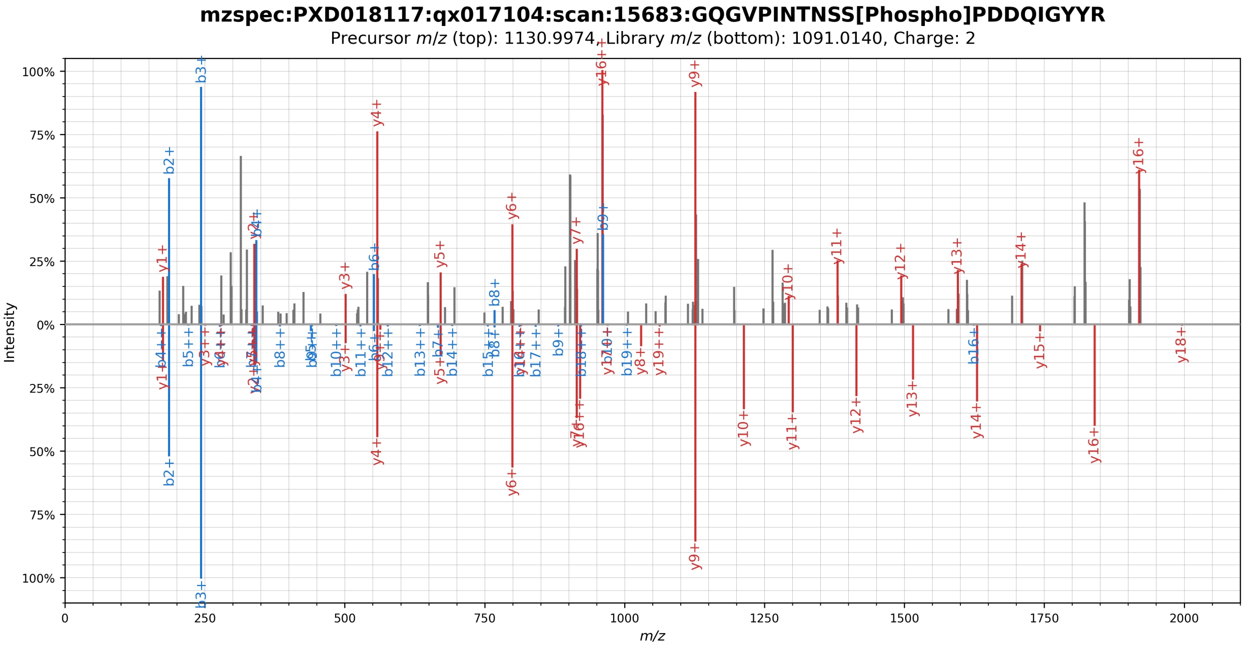

Fig. S5. Mirror plot showing the spectral library match (bottom) of a phosphorylated peptide originating from the SARS-CoV-2 N protein (top). The phosphorylation was localized to residue S79 by visual inspection.

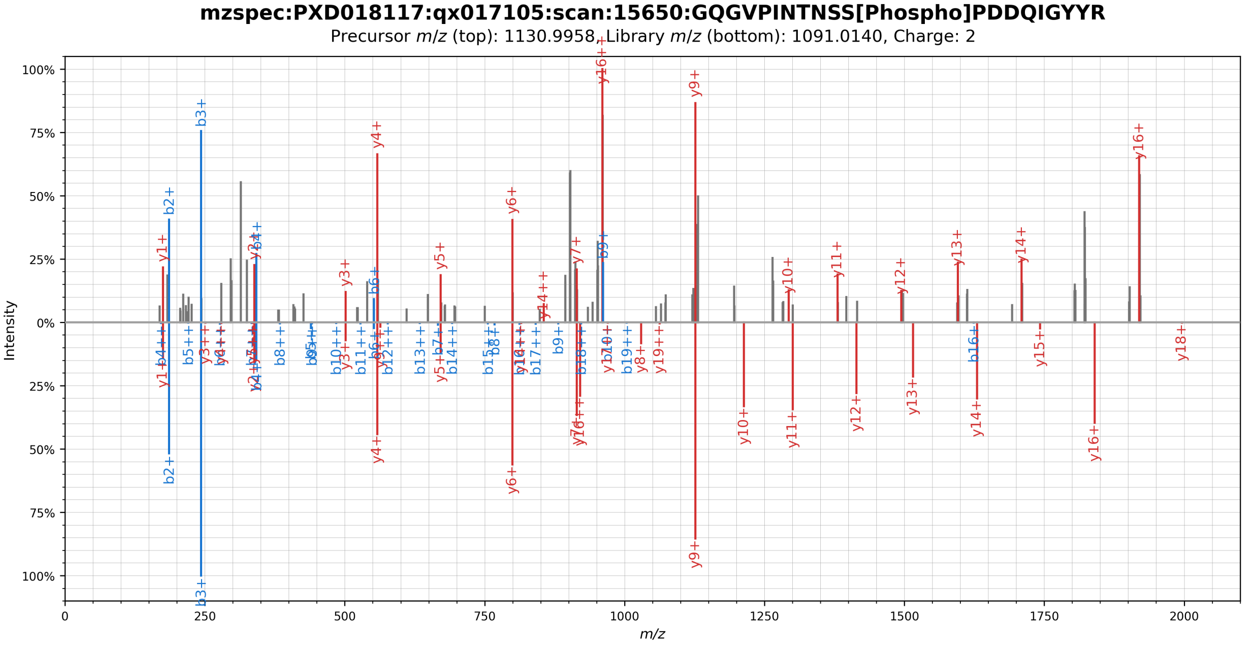

Fig. S6. Mirror plot showing the spectral library match (bottom) of a phosphorylated peptide originating from the SARS-CoV-2 N protein (top). The phosphorylation was localized to residue S79 by visual inspection.

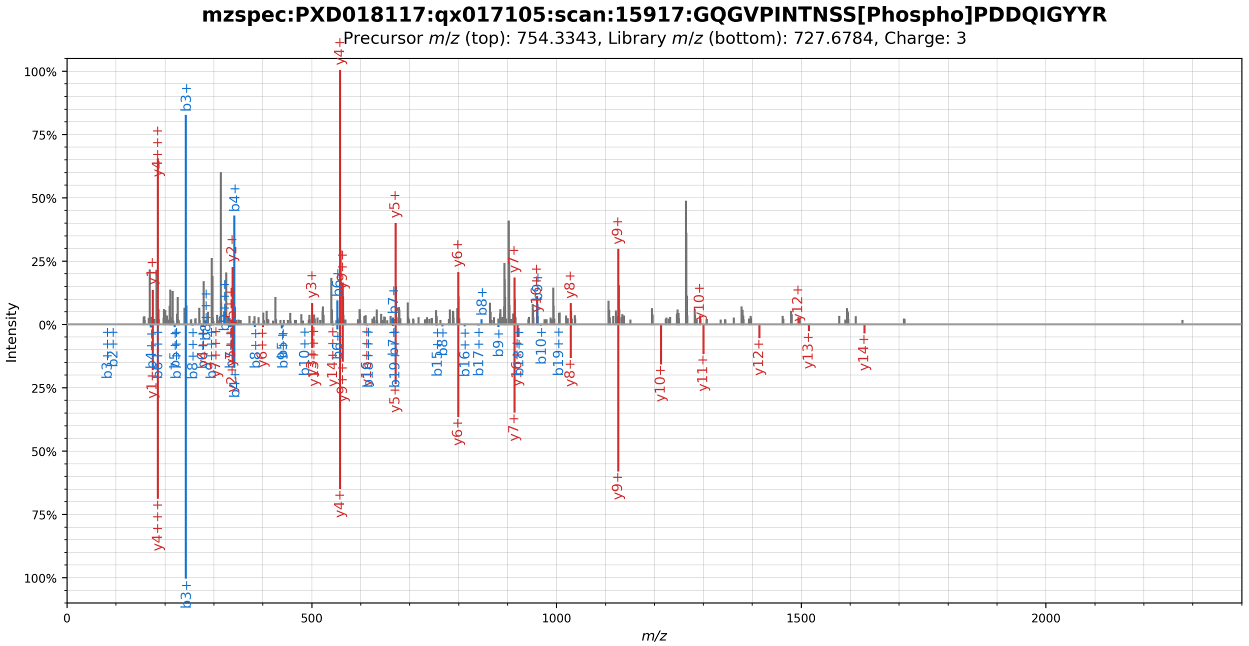

Fig. S7. Mirror plot showing the spectral library match (bottom) of a phosphorylated peptide originating from the SARS-CoV-2 N protein (top). The phosphorylation was localized to residue S79 by visual inspection.

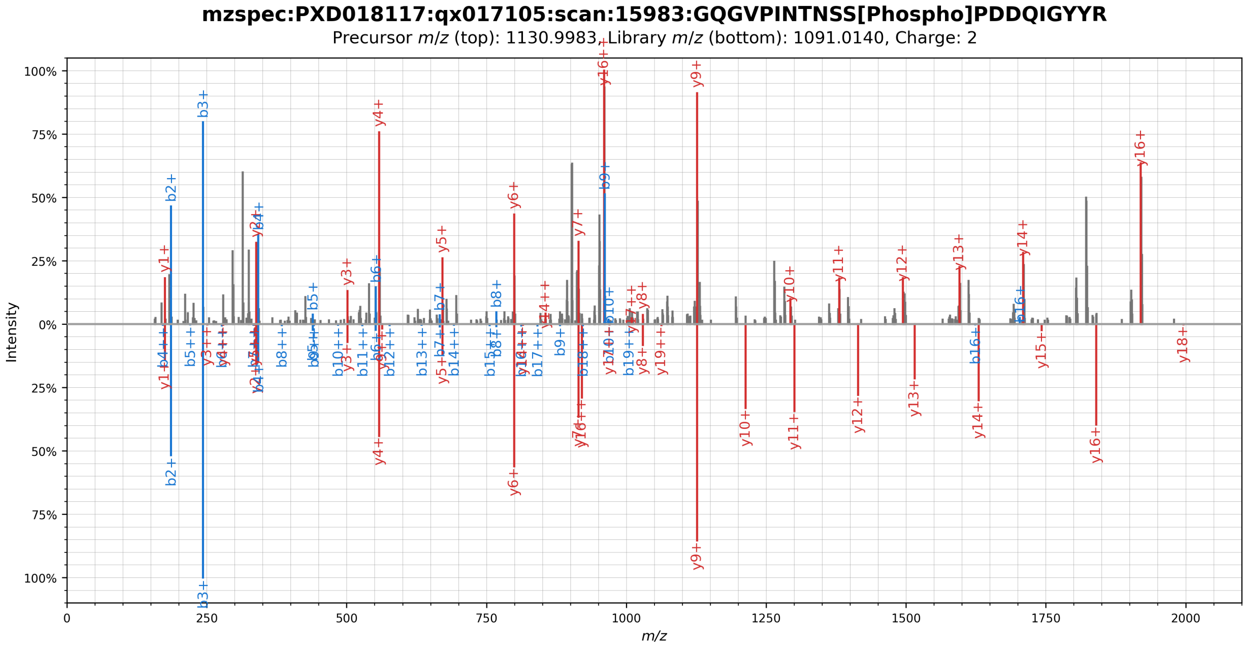

Fig. S8. Mirror plot showing the spectral library match (bottom) of a phosphorylated peptide originating from the SARS-CoV-2 N protein (top). The phosphorylation was localized to residue S79 by visual inspection.

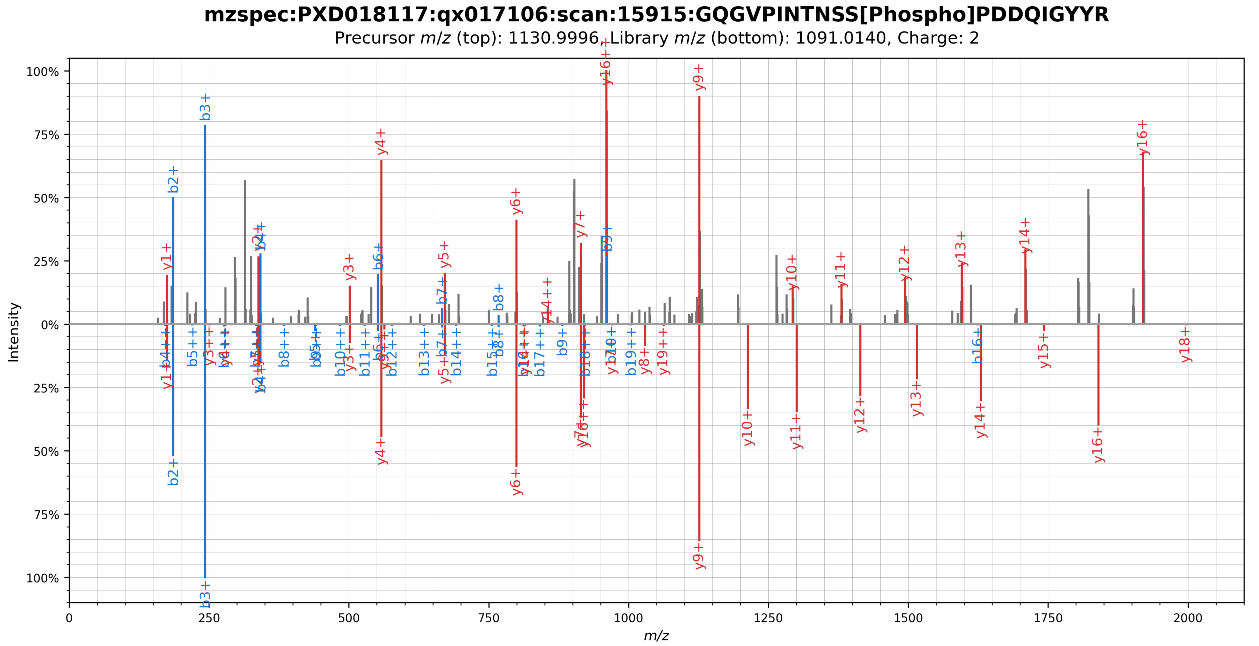

Fig. S9. Mirror plot showing the spectral library match (bottom) of a phosphorylated peptide originating from the SARS-CoV-2 N protein (top). The phosphorylation was localized to residue S79 by visual inspection.

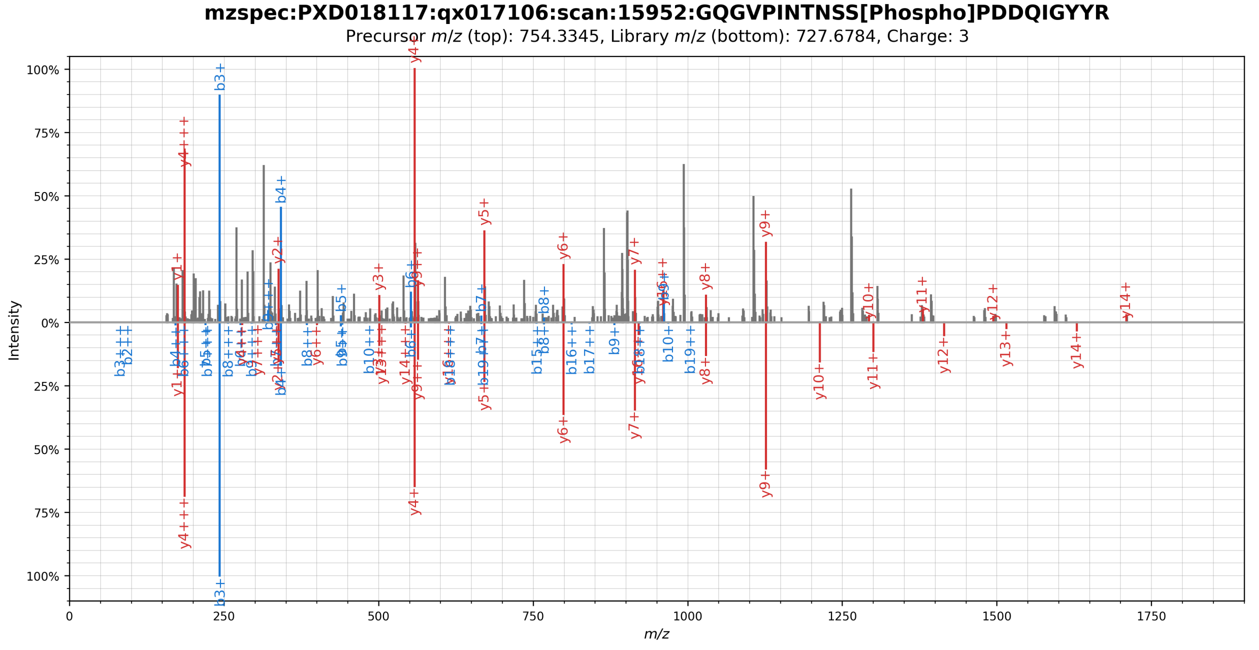

Fig. S10. Mirror plot showing the spectral library match (bottom) of a phosphorylated peptide originating from the SARS-CoV-2 N protein (top). The phosphorylation was localized to residue S79 by visual inspection.

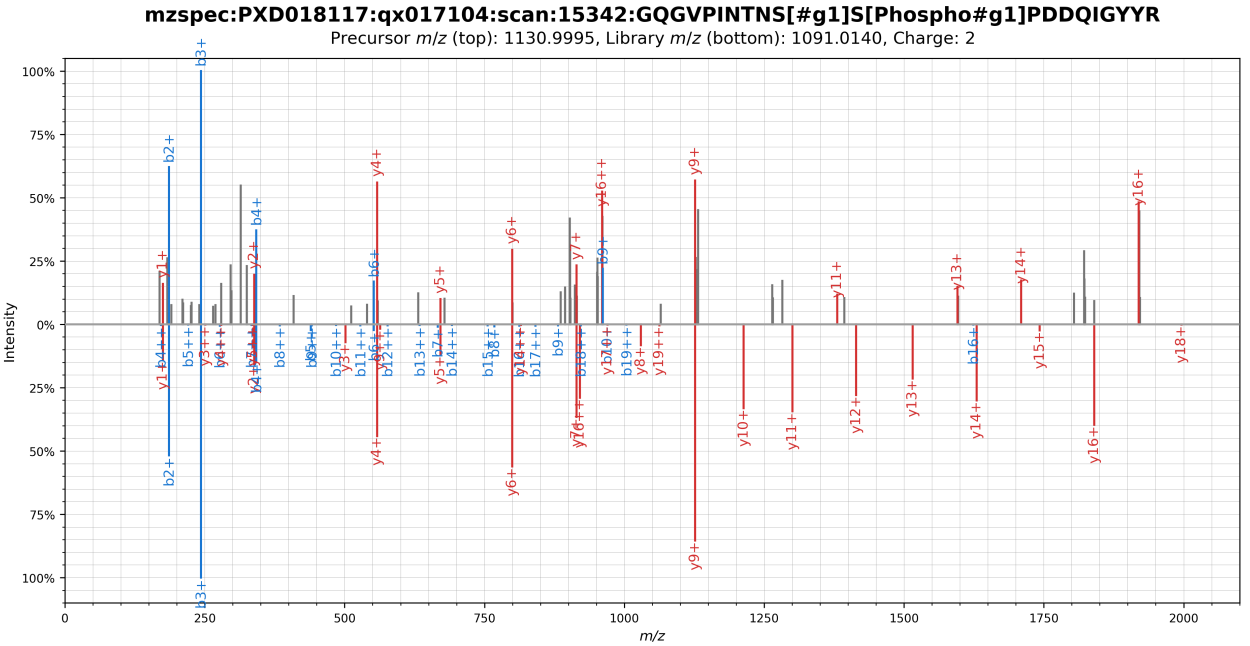

Fig. S11. Mirror plot showing the spectral library match (bottom) of a phosphorylated peptide originating from the SARS-CoV-2 N protein (top). The phosphorylation was localized to residue S78/S79 by visual inspection.

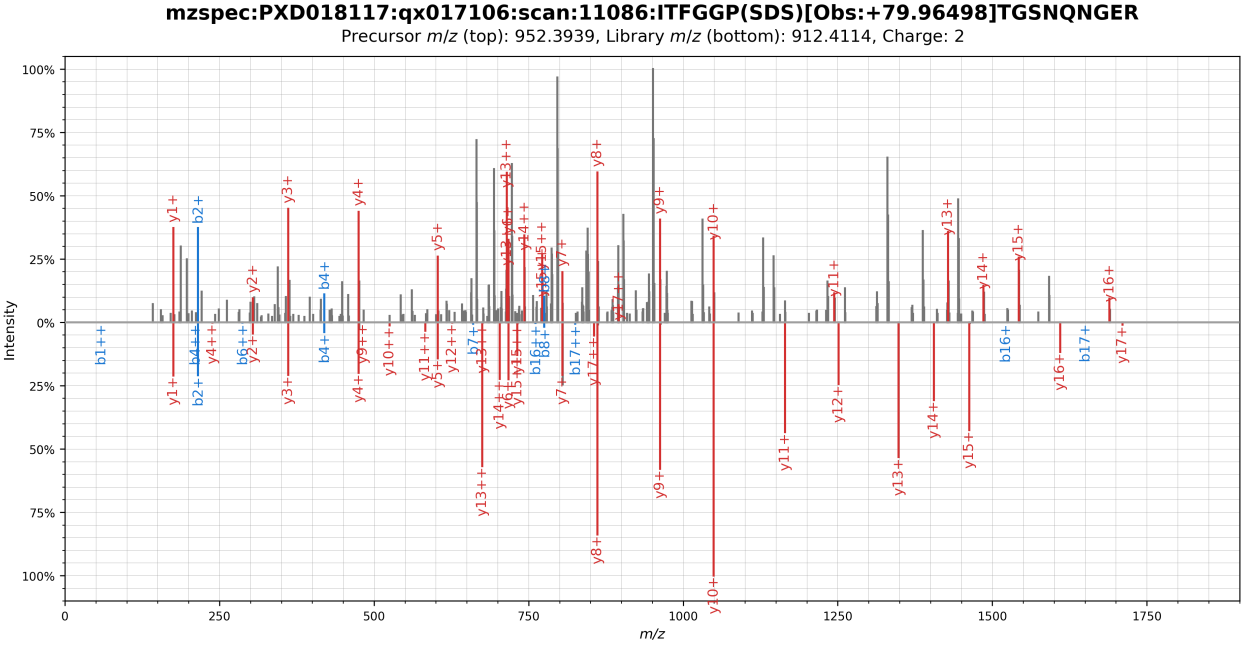

Fig. S12. Mirror plot showing the spectral library match (bottom) of a phosphorylated peptide originating from the SARS-CoV-2 N protein (top). The phosphorylation was localized to residue S21/S23 by visual inspection.

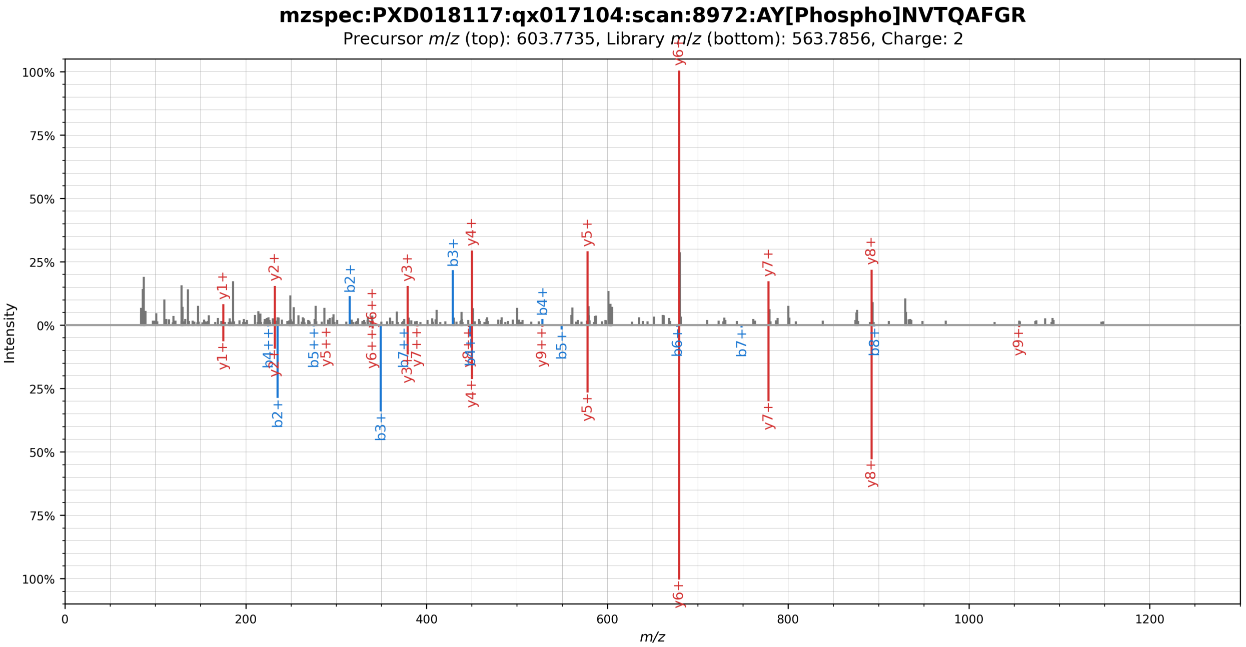

Fig. S13. Mirror plot showing the spectral library match (bottom) of a phosphorylated peptide originating from the SARS-CoV-2 N protein (top). The phosphorylation was localized to residue Y268 by visual inspection.

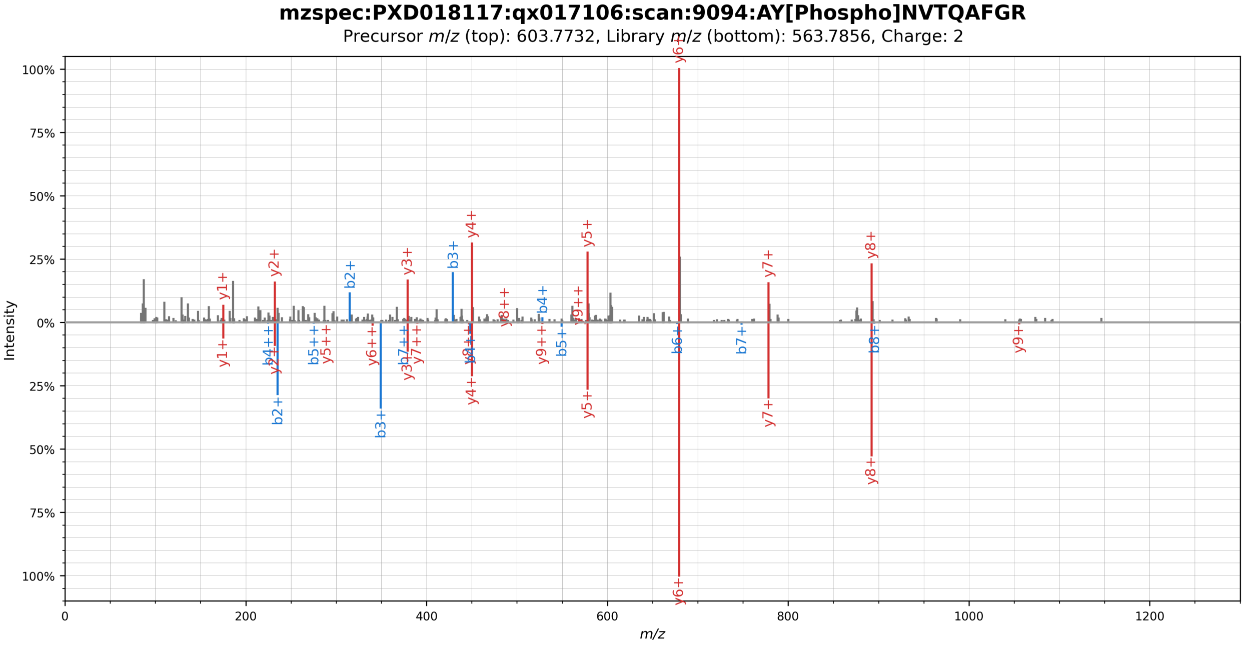

Fig. S14. Mirror plot showing the spectral library match (bottom) of a phosphorylated peptide originating from the SARS-CoV-2 N protein (top). The phosphorylation was localized to residue Y268 by visual inspection.

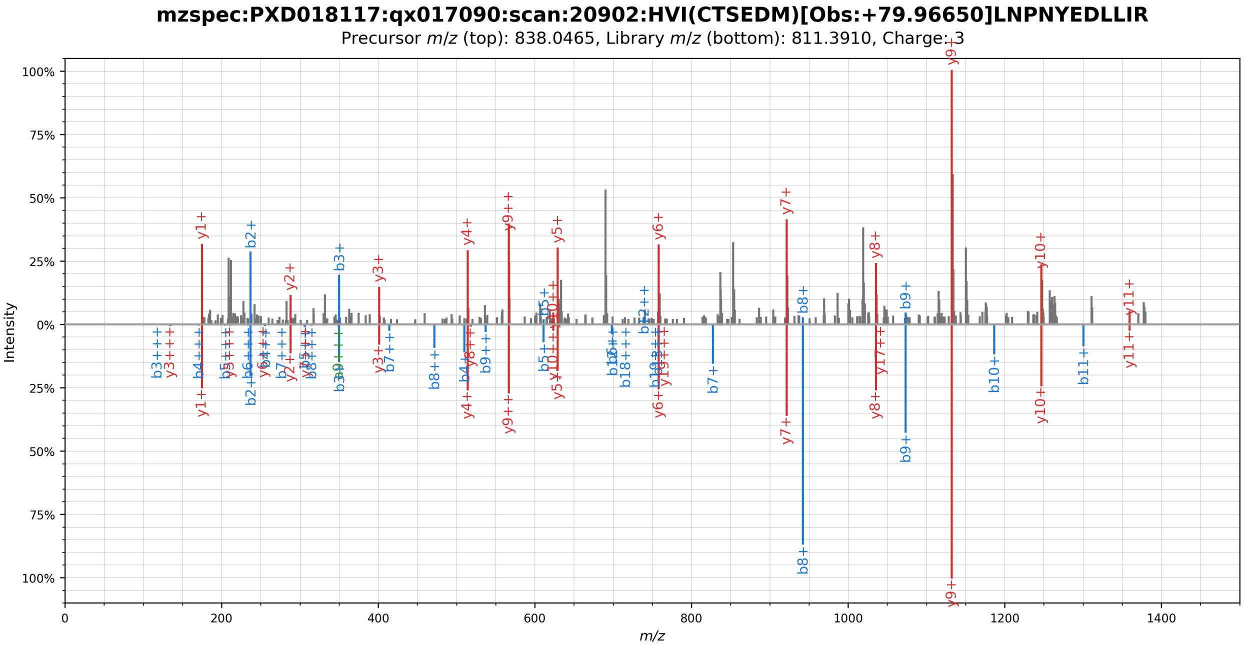

Fig. S15. Mirror plot showing the spectral library match (bottom) of a phosphorylated peptide originating from the SARS-CoV-2 Nsp5 protein (top). The phosphorylation was localized to residue T46/S47 by visual inspection.

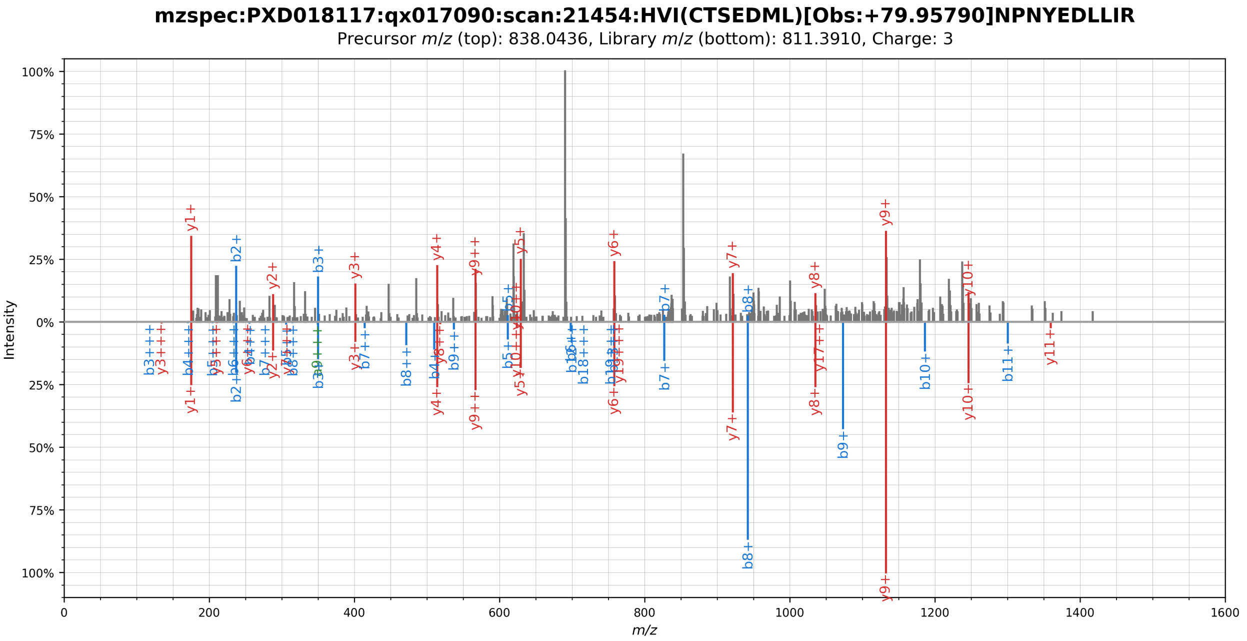

Fig. S16. Mirror plot showing the spectral library match (bottom) of a phosphorylated peptide originating from the SARS-CoV-2 Nsp5 protein (top). The phosphorylation was localized to residue T46/S47 by visual inspection.

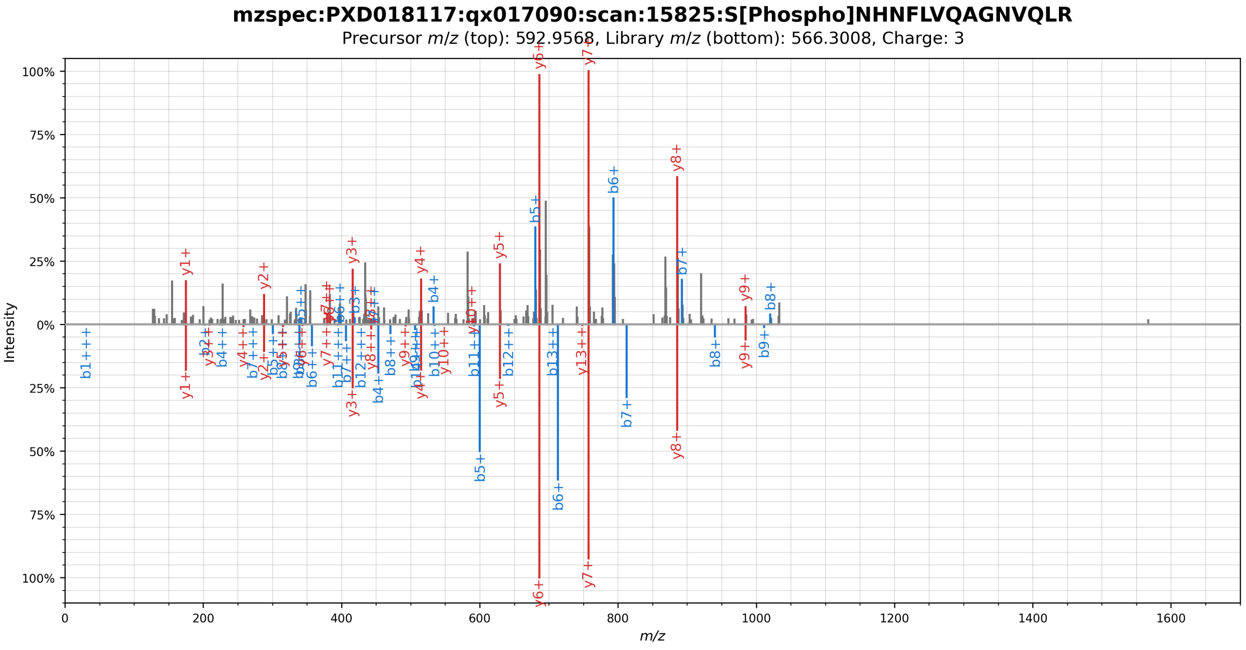

Fig. S17. Mirror plot showing the spectral library match (bottom) of a phosphorylated peptide originating from the SARS-CoV-2 Nsp5 protein (top). The phosphorylation was localized to residue S63 by visual inspection.

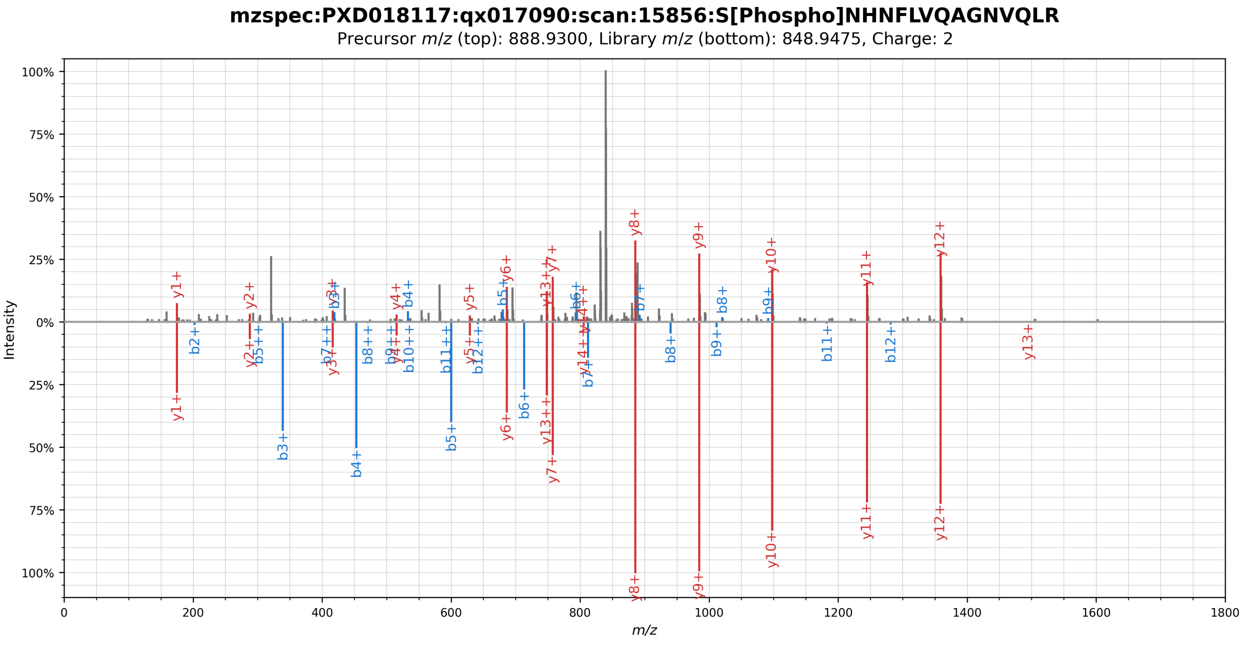

Fig. S18. Mirror plot showing the spectral library match (bottom) of a phosphorylated peptide originating from the SARS-CoV-2 Nsp5 protein (top). The phosphorylation was localized to residue S63 by visual inspection.

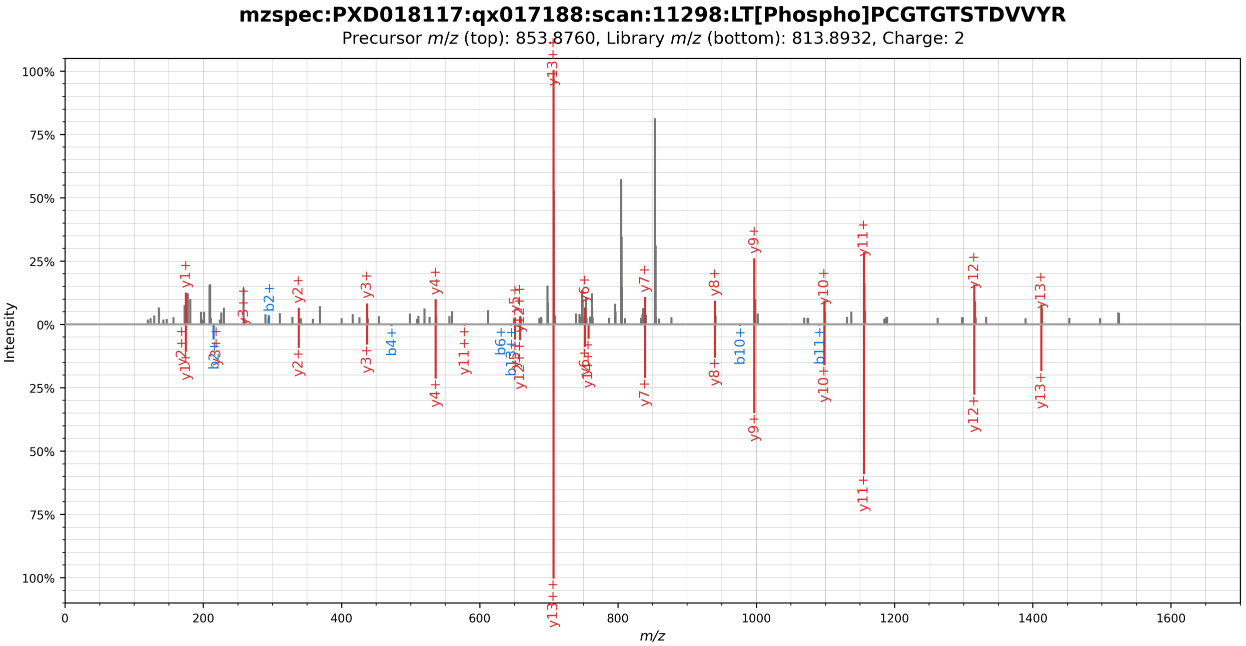

Fig. S19. Mirror plot showing the spectral library match (bottom) of a phosphorylated peptide originating from the SARS-CoV-2 Nsp12 protein (top). The phosphorylation was localized to residue T21 by visual inspection.

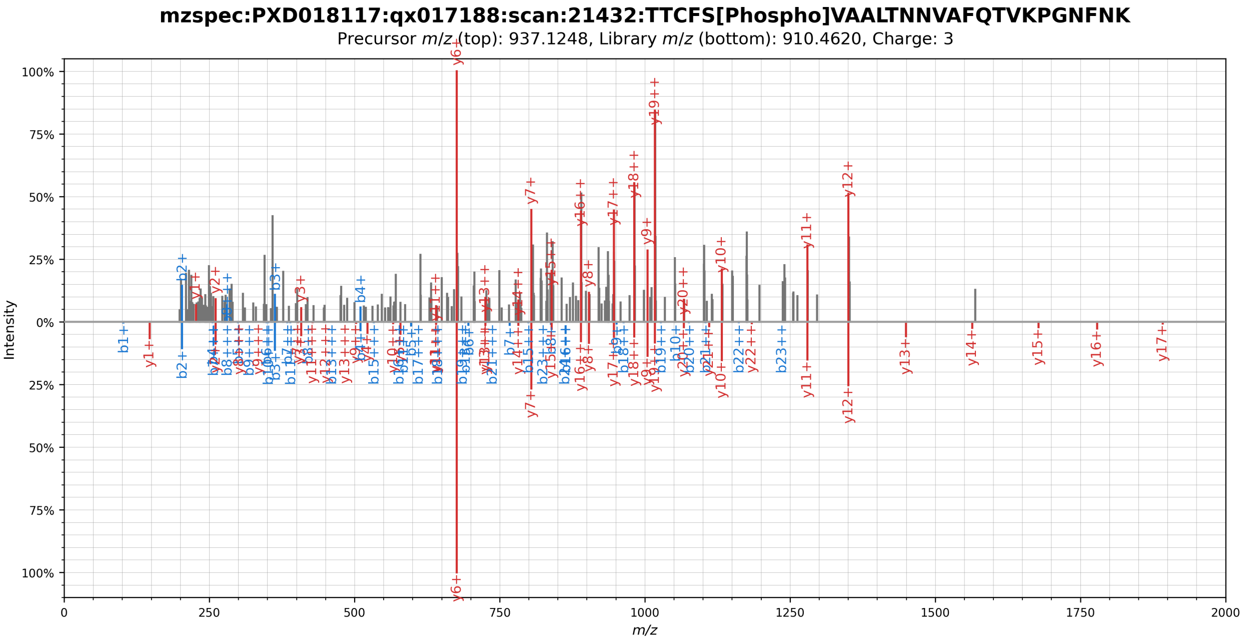

Fig. S20. Mirror plot showing the spectral library match (bottom) of a phosphorylated peptide originating from the SARS-CoV-2 Nsp12 protein (top). The phosphorylation was localized to residue S398 by visual inspection.

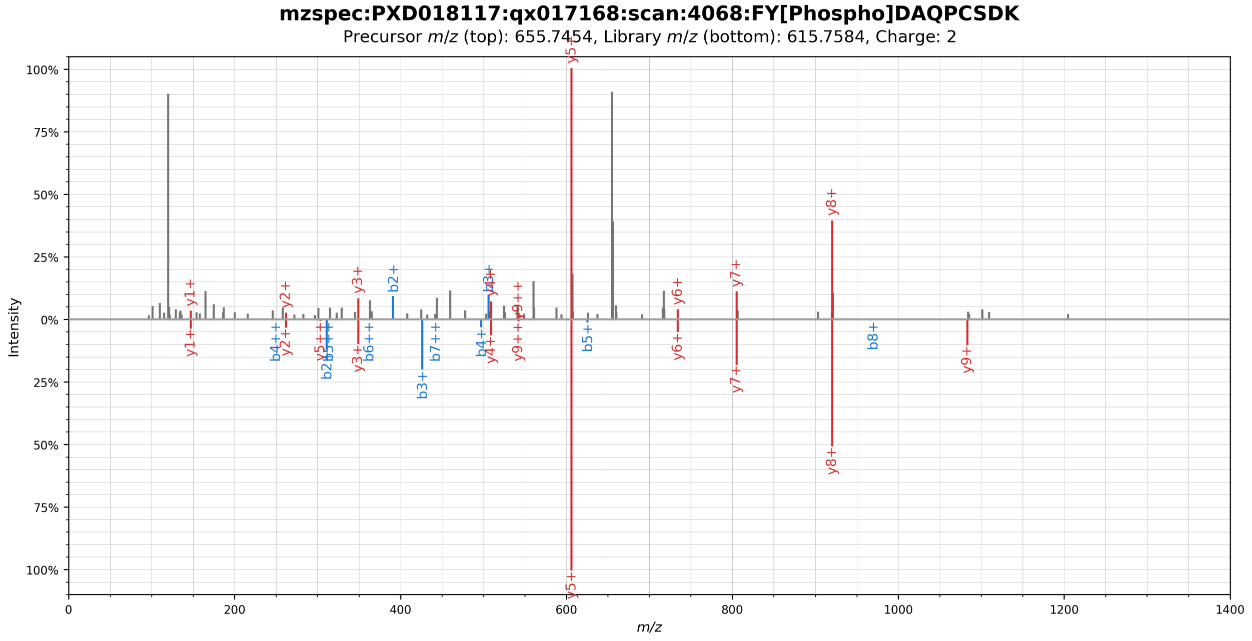

Fig. S21. Mirror plot showing the spectral library match (bottom) of a phosphorylated peptide originating from the SARS-CoV-2 Nsp14 protein (top). The phosphorylation was localized to residue Y351 by visual inspection.

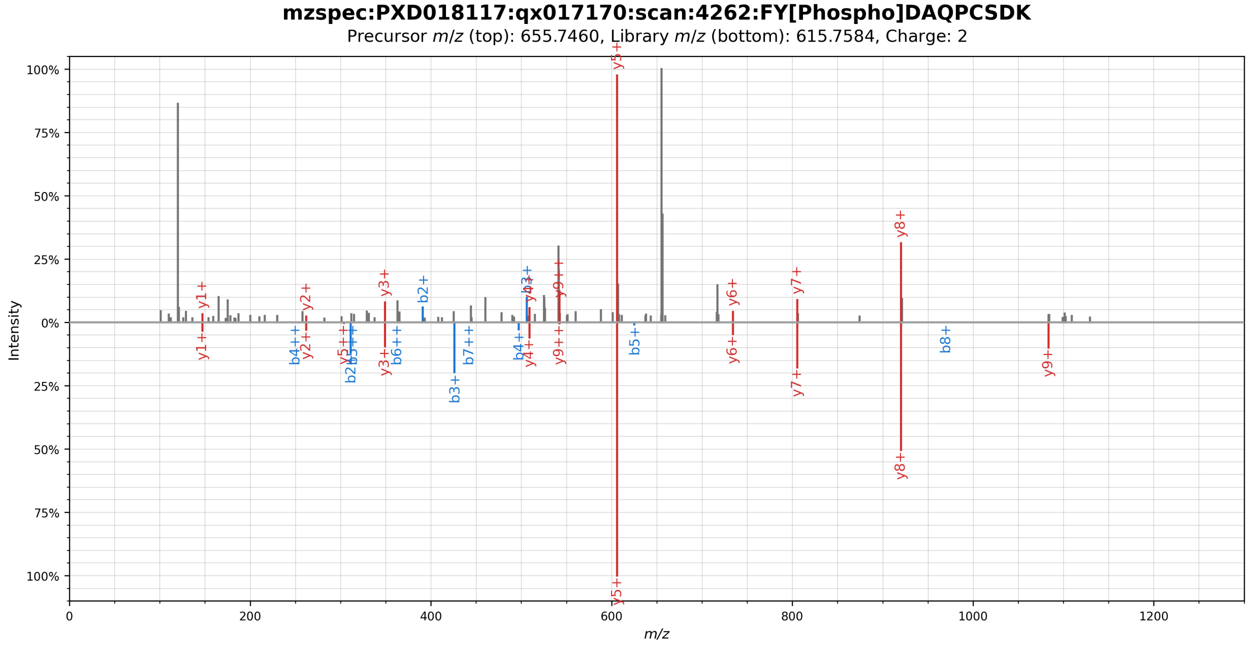

Fig. S22. Mirror plot showing the spectral library match (bottom) of a phosphorylated peptide originating from the SARS-CoV-2 Nsp14 protein (top). The phosphorylation was localized to residue Y351 by visual inspection.

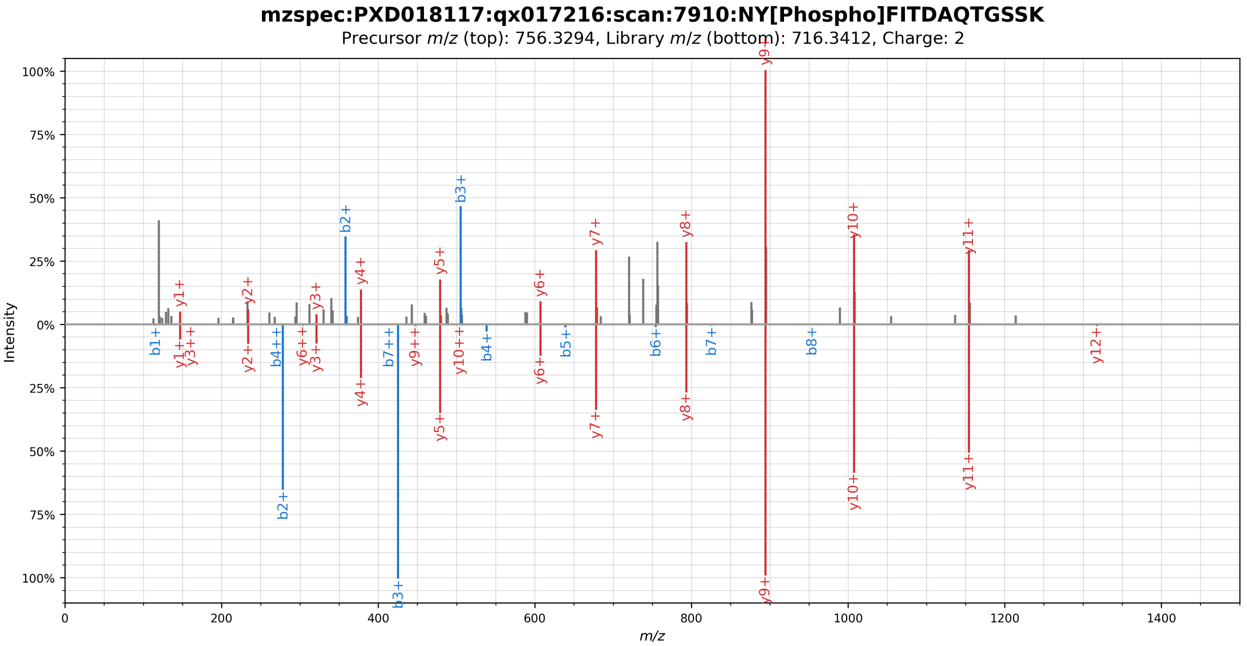

Fig. S23. Mirror plot showing the spectral library match (bottom) of a phosphorylated peptide originating from the SARS-CoV-2 Nsp15 protein (top). The phosphorylation was localized to residue Y279 by visual inspection.

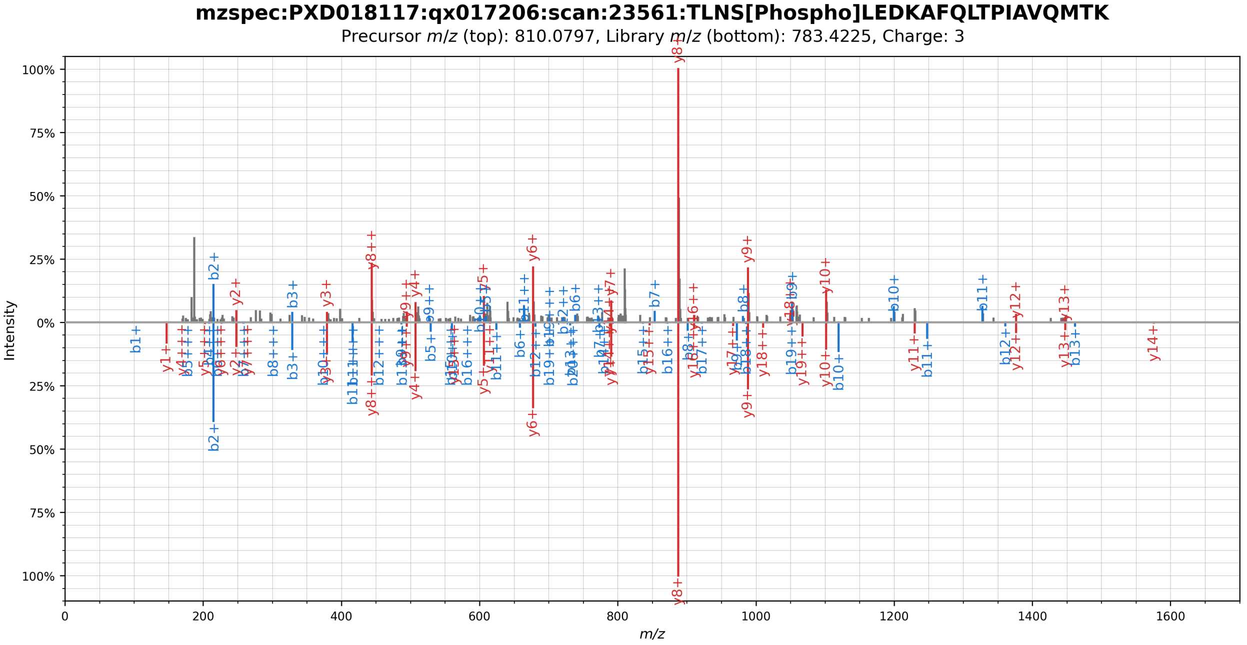

Fig. S24. Mirror plot showing the spectral library match (bottom) of a phosphorylated peptide originating from the SARS-CoV-2 Orf9b protein (top). The phosphorylation was localized to residue S63 by visual inspection.

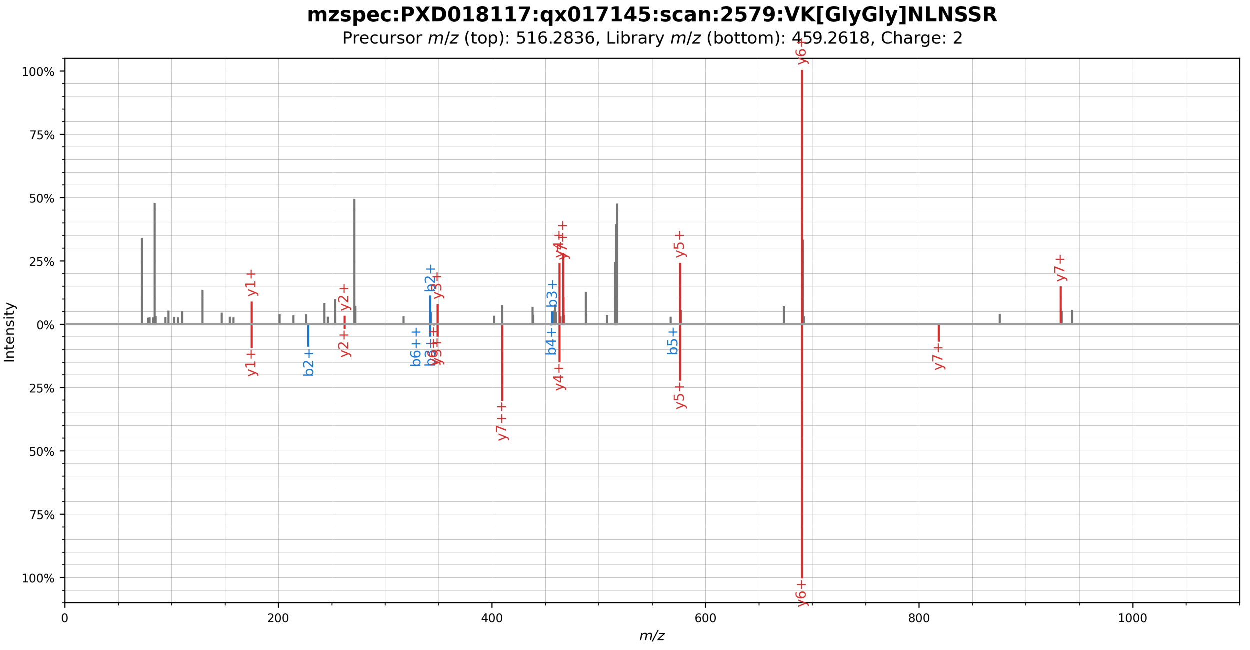

Fig. S25. Mirror plot showing the spectral library match (bottom) of a ubiquitylated peptide originating from the SARS-CoV-2 E protein (top). The phosphorylation was localized to residue K63 by visual inspection.

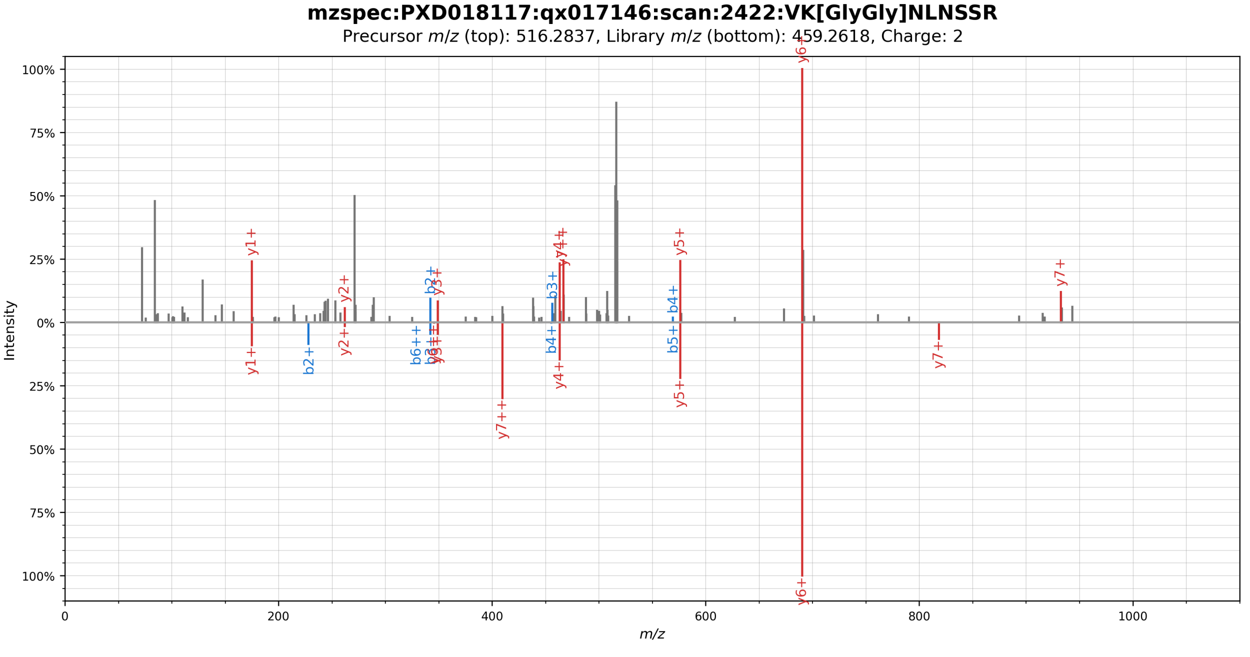

Fig. S26. Mirror plot showing the spectral library match (bottom) of a ubiquitylated peptide originating from the SARS-CoV-2 E protein (top). The phosphorylation was localized to residue K63 by visual inspection.

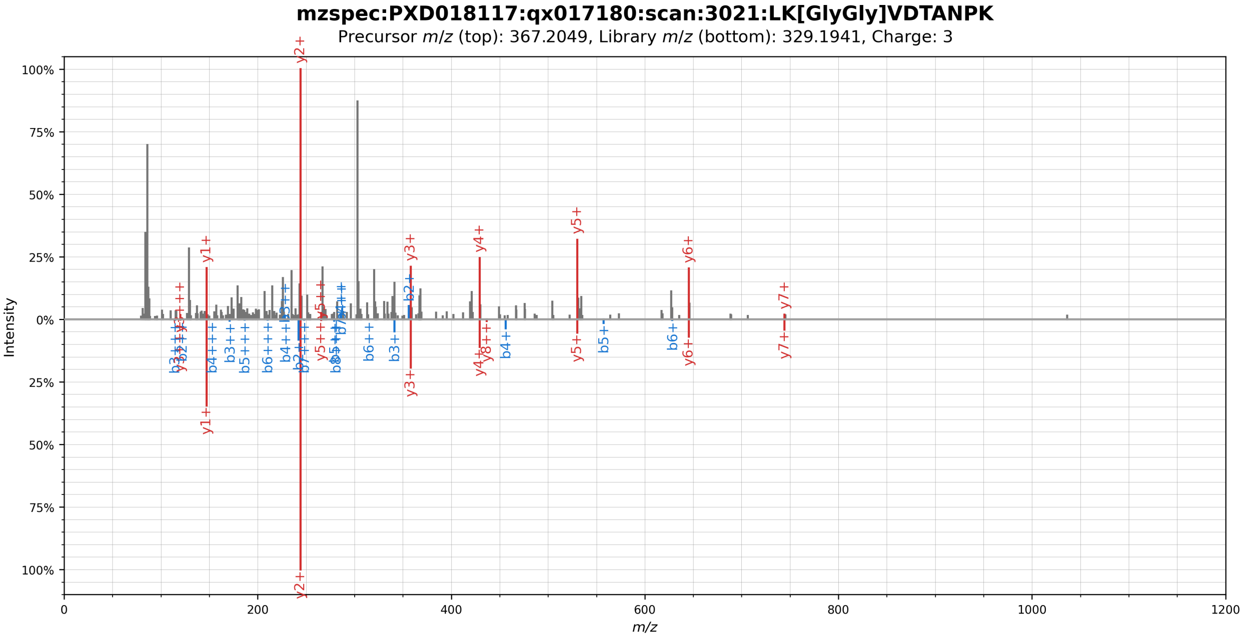

Fig. S27. Mirror plot showing the spectral library match (bottom) of a ubiquitylated peptide originating from the SARS-CoV-2 Nsp5(C145A) protein (top). The phosphorylation was localized to residue K90 by visual inspection.

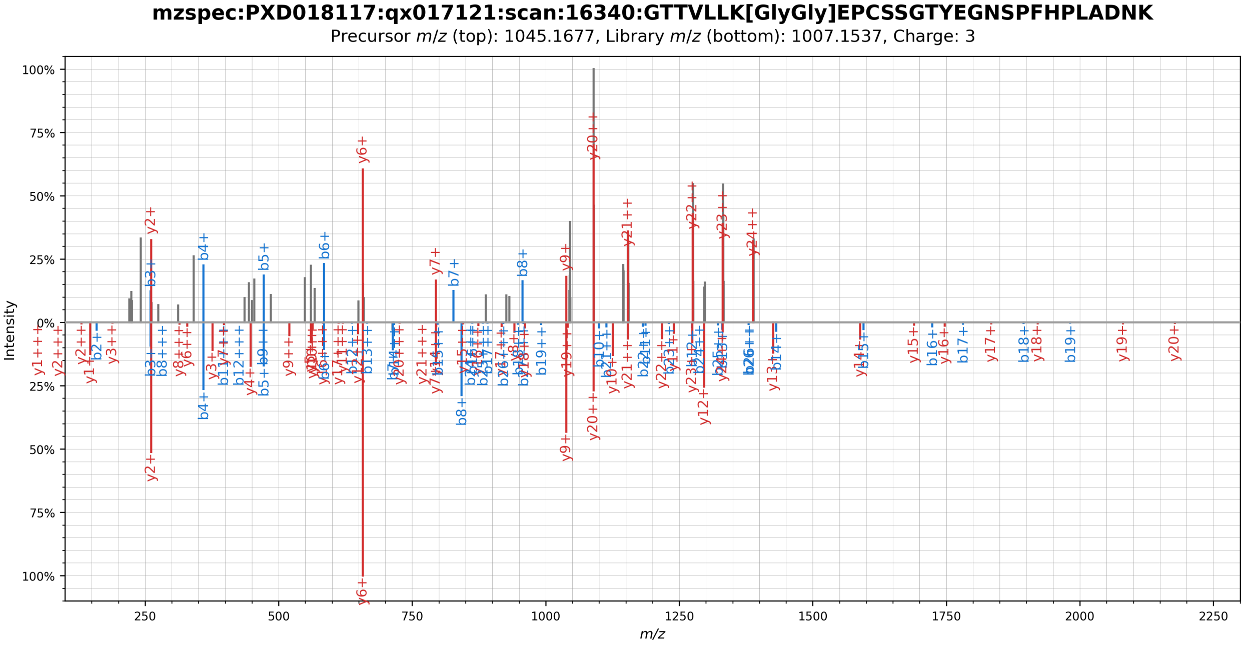

Fig. S28. Mirror plot showing the spectral library match (bottom) of a ubiquitylated peptide originating from the SARS-CoV-2 Orf7a protein (top). The phosphorylation was localized to residue K32 by visual inspection.

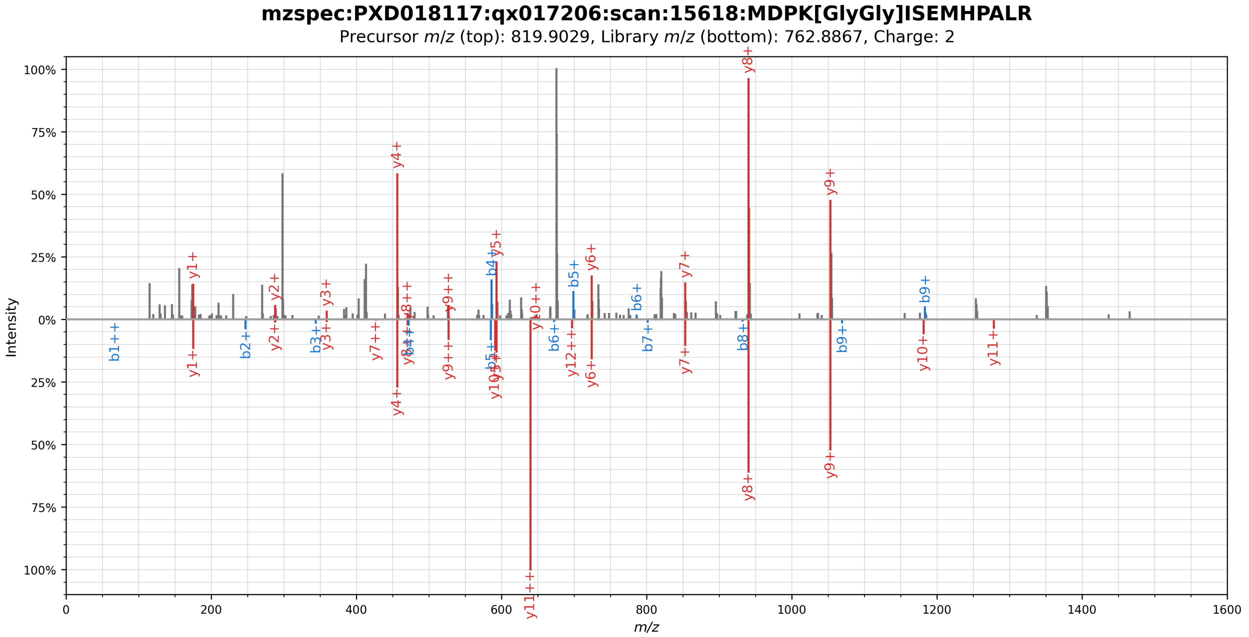

Fig. S29. Mirror plot showing the spectral library match (bottom) of a ubiquitylated peptide originating from the SARS-CoV-2 Orf9b protein (top). The phosphorylation was localized to residue K4 by visual inspection.

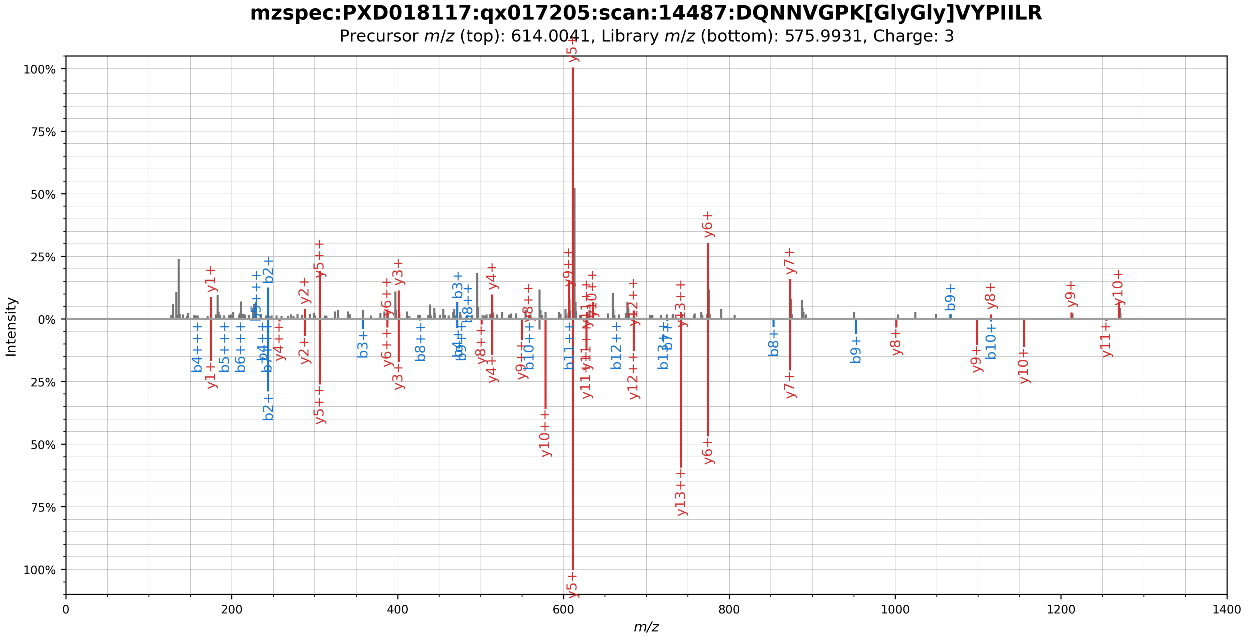

Fig. S30. Mirror plot showing the spectral library match (bottom) of a ubiquitylated peptide originating from the SARS-CoV-2 Orf9b protein (top). The phosphorylation was localized to residue K40 by visual inspection.

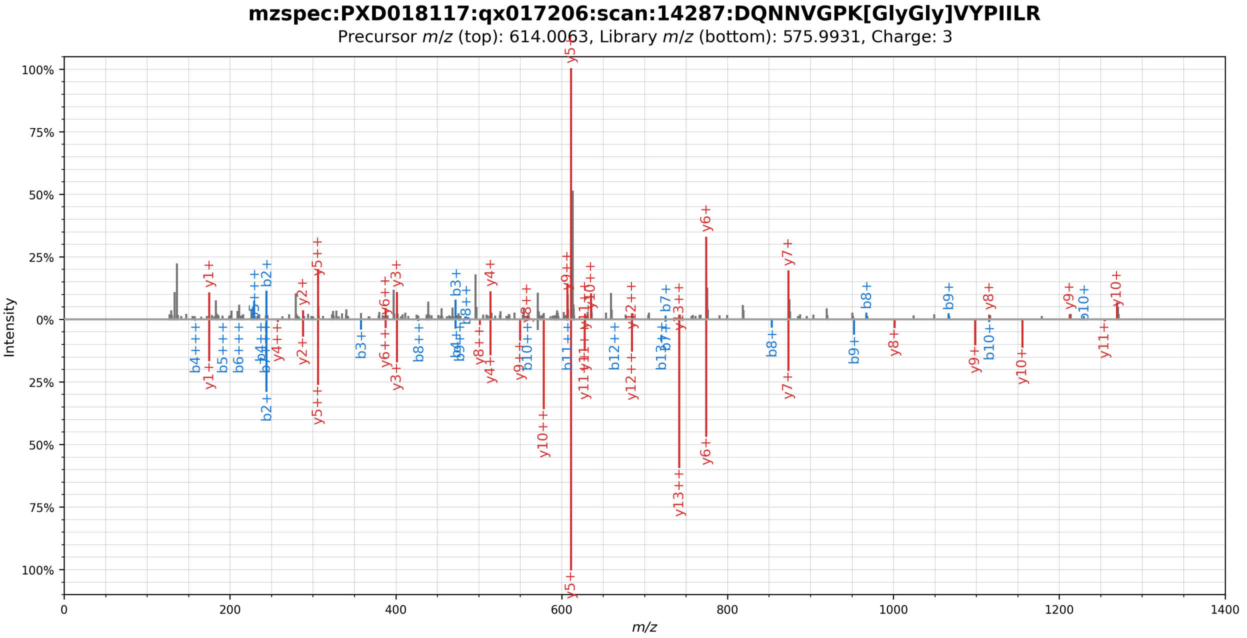

Fig. S31. Mirror plot showing the spectral library match (bottom) of a ubiquitylated peptide originating from the SARS-CoV-2 Orf9b protein (top). The phosphorylation was localized to residue K40 by visual inspection.

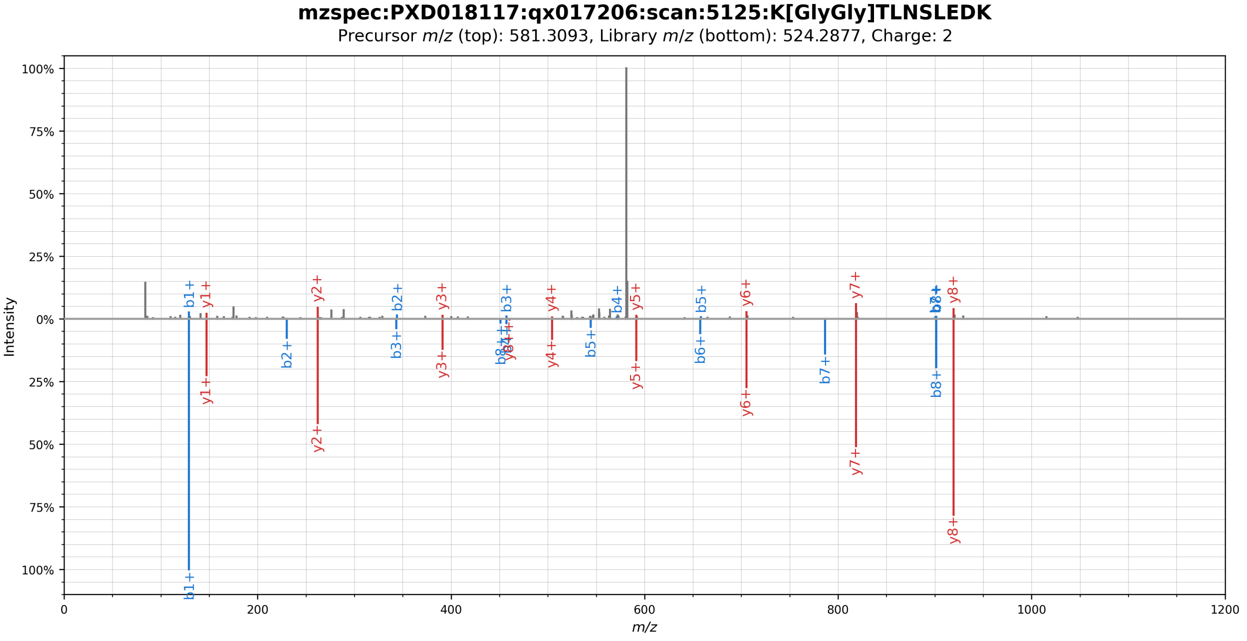

Fig. S32. Mirror plot showing the spectral library match (bottom) of a ubiquitylated peptide originating from the SARS-CoV-2 Orf9b protein (top). The phosphorylation was localized to residue K59 by visual inspection.

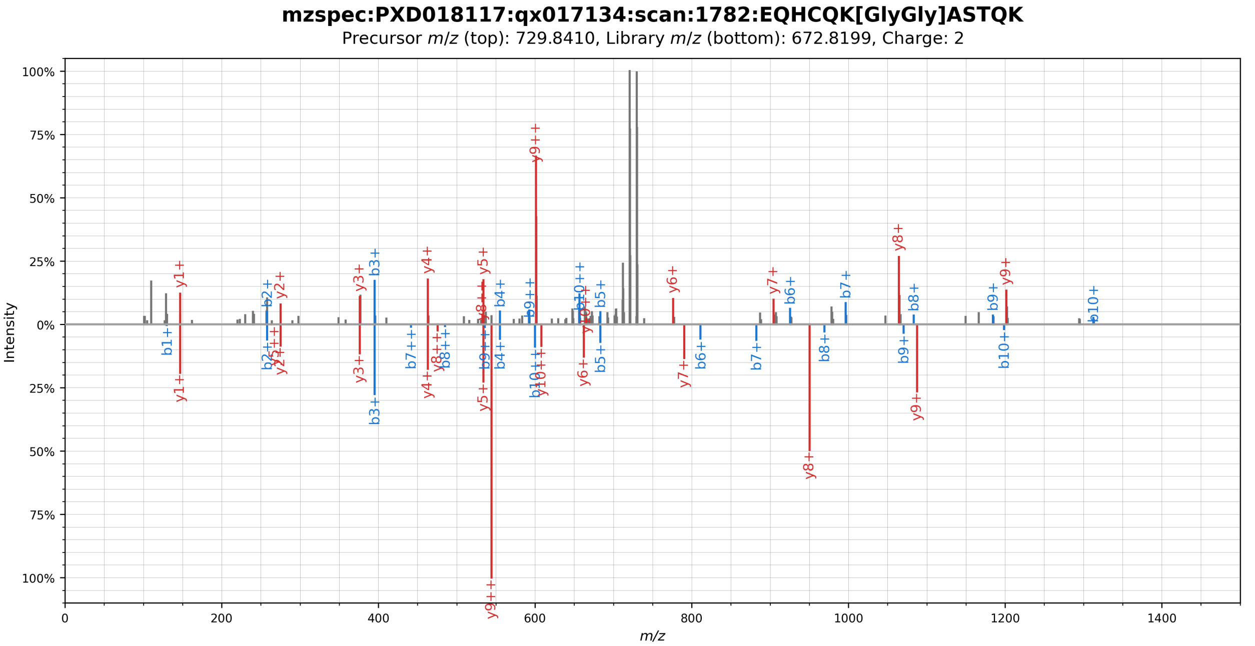

Fig. S33. Mirror plot showing the spectral library match (bottom) of a ubiquitylated peptide originating from the SARS-CoV-2 Orf9c protein (top). The phosphorylation was localized to residue K16 by visual inspection.

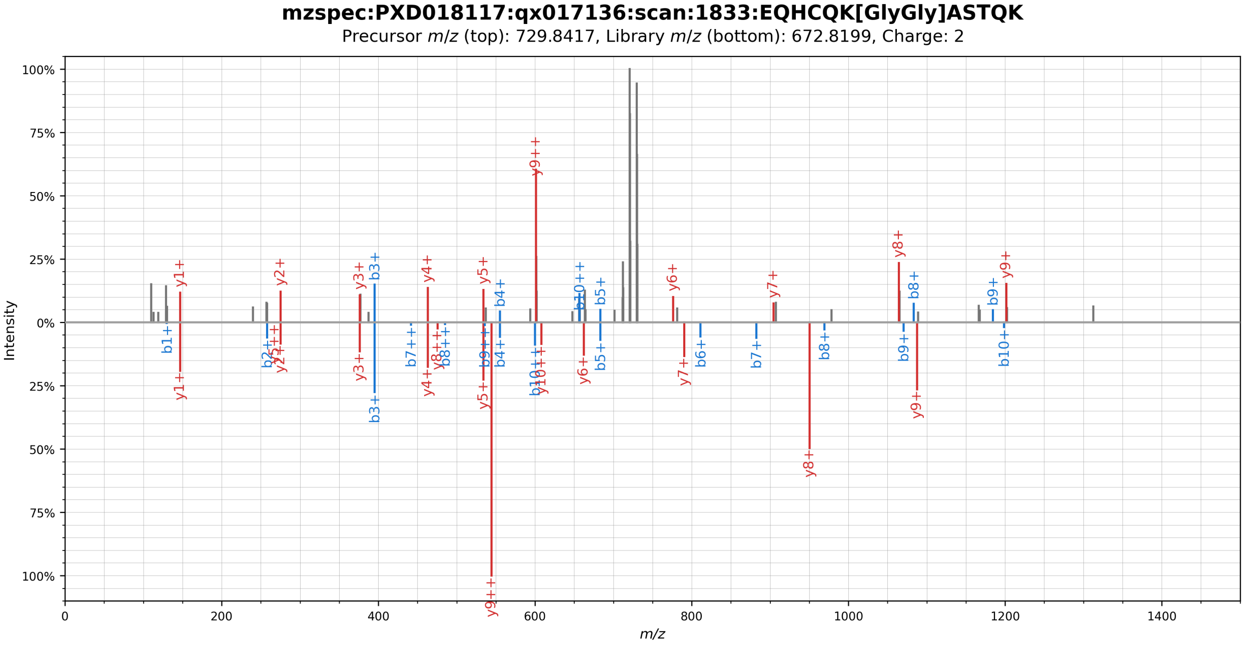

Fig. S34. Mirror plot showing the spectral library match (bottom) of a ubiquitylated peptide originating from the SARS-CoV-2 Orf9c protein (top). The phosphorylation was localized to residue K16 by visual inspection.

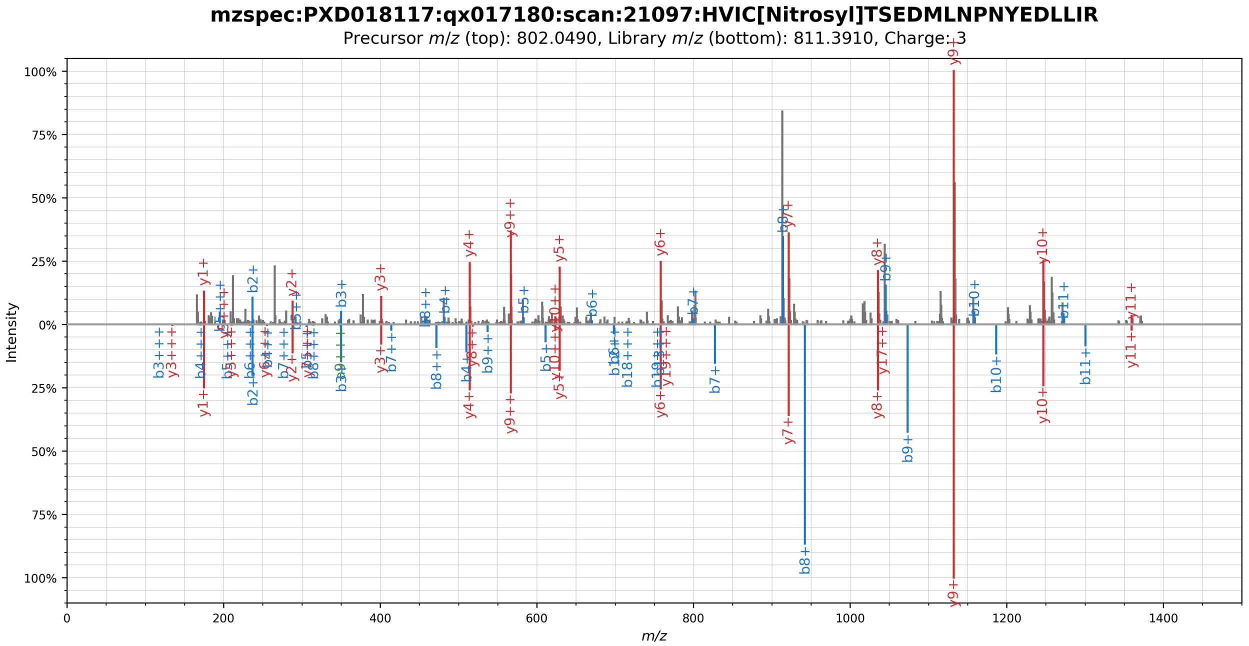

Fig. S35. Mirror plot showing the spectral library match (bottom) of a S-nitrosylated peptide originating from the SARS-CoV-2 Nsp5(C145A) protein (top). The S-nitrosylation was localized to residue C45 by visual inspection.

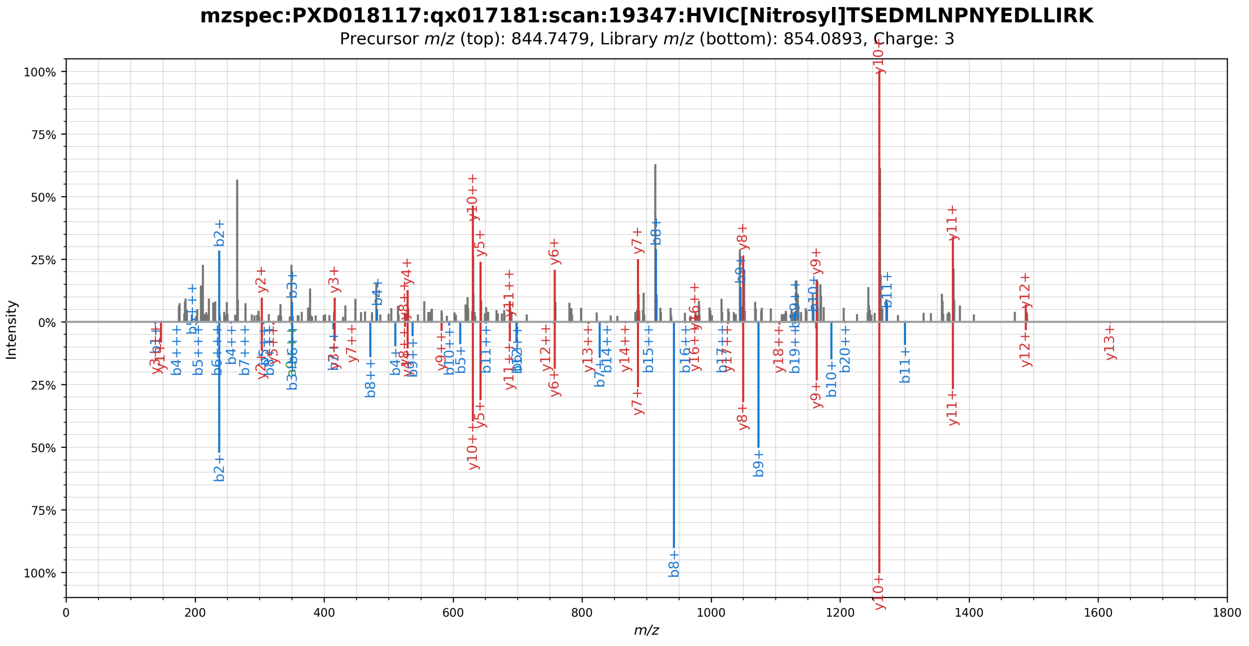

Fig. S36. Mirror plot showing the spectral library match (bottom) of a S-nitrosylated peptide originating from the SARS-CoV-2 Nsp5(C145A) protein (top). The S-nitrosylation was localized to residue C45 by visual inspection.

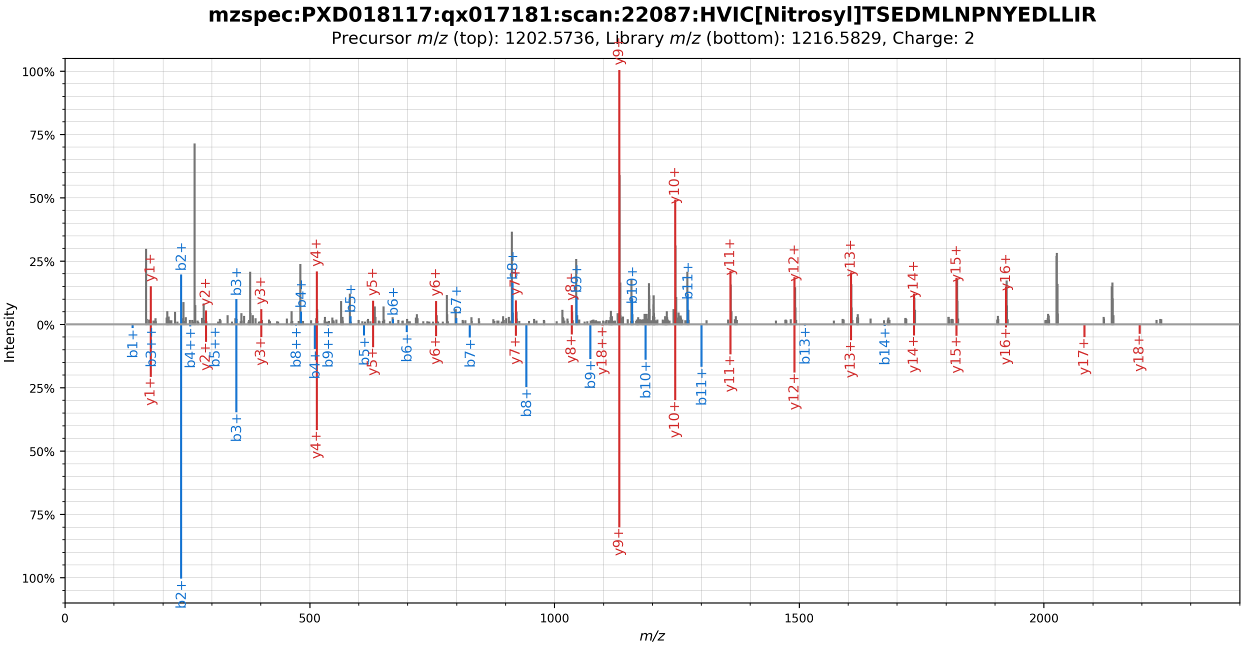

Fig. S37. Mirror plot showing the spectral library match (bottom) of a S-nitrosylated peptide originating from the SARS-CoV-2 Nsp5(C145A) protein (top). The S-nitrosylation was localized to residue C45 by visual inspection.

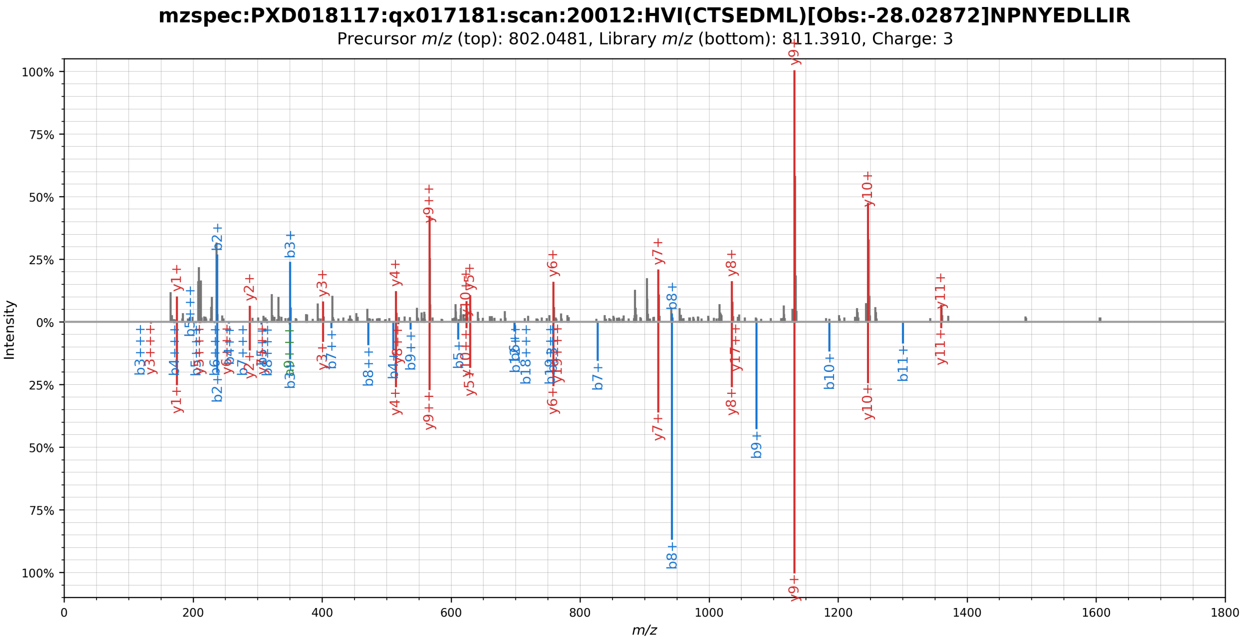

Fig. S38. Mirror plot showing the spectral library match (bottom) of a possibly S-nitrosylated peptide originating from the SARS-CoV-2 Nsp5(C145A) protein (top). The mass shift could not be localized by visual inspection.

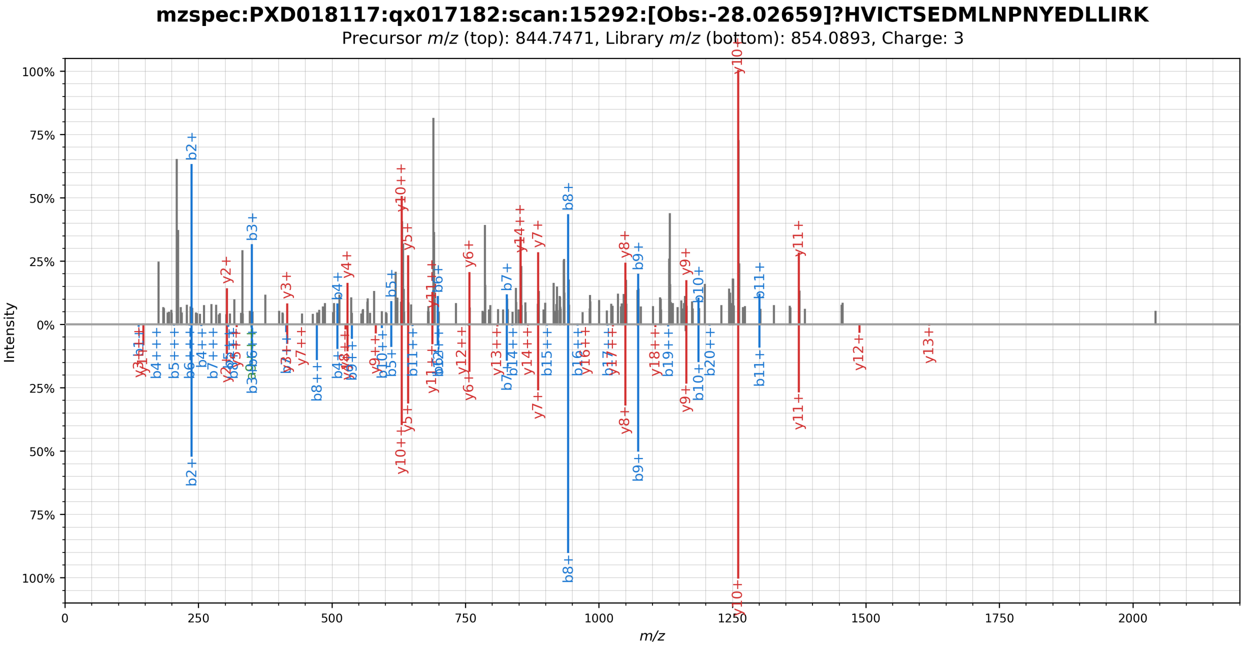

Fig. S39. Mirror plot showing the spectral library match (bottom) of a possibly S-nitrosylated peptide originating from the SARS-CoV-2 Nsp5(C145A) protein (top). The mass shift could not be localized by visual inspection.

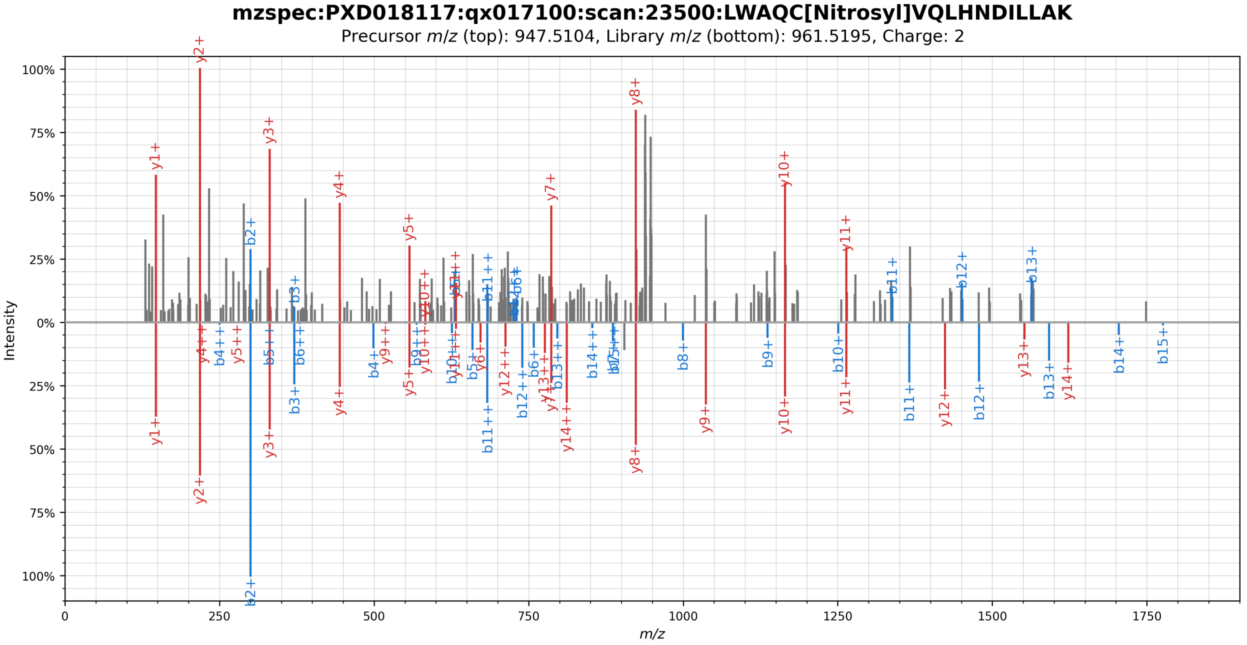

Fig. S40. Mirror plot showing the spectral library match (bottom) of a S-nitrosylated peptide originating from the SARS-CoV-2 Nsp7 protein (top). The S-nitrosylation was localized to residue C33 by visual inspection.

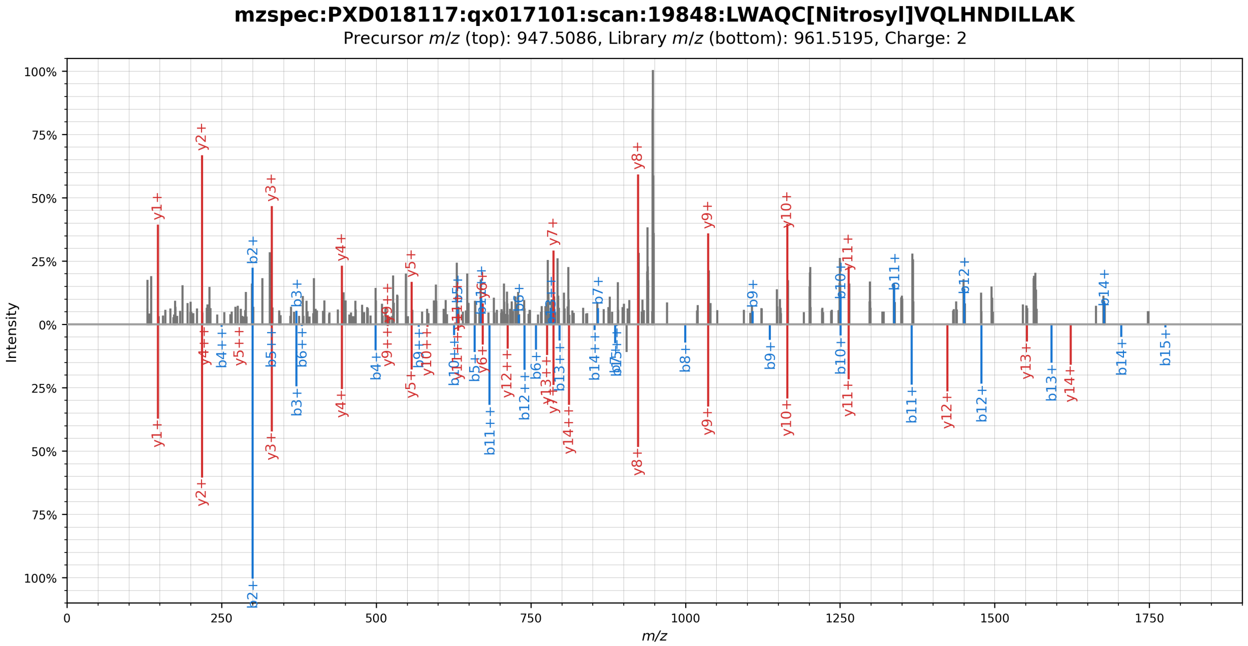

Fig. S41. Mirror plot showing the spectral library match (bottom) of a S-nitrosylated peptide originating from the SARS-CoV-2 Nsp7 protein (top). The S-nitrosylation was localized to residue C33 by visual inspection.

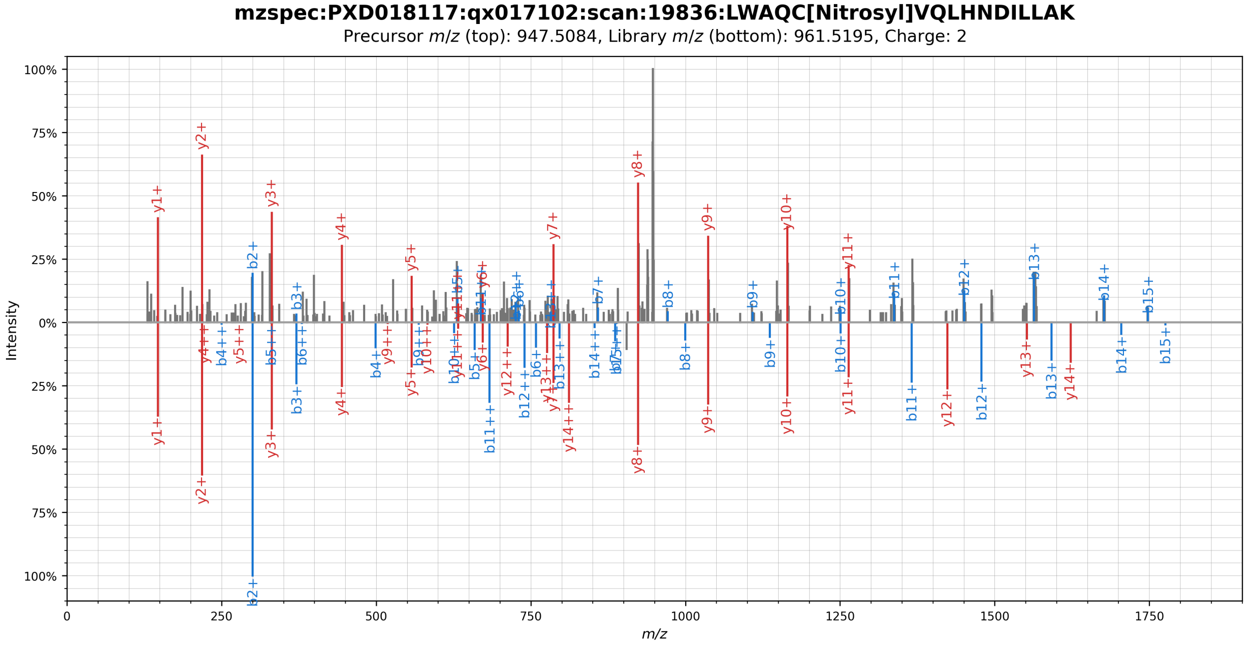

Fig. S42. Mirror plot showing the spectral library match (bottom) of a S-nitrosylated peptide originating from the SARS-CoV-2 Nsp7 protein (top). The S-nitrosylation was localized to residue C33 by visual inspection.

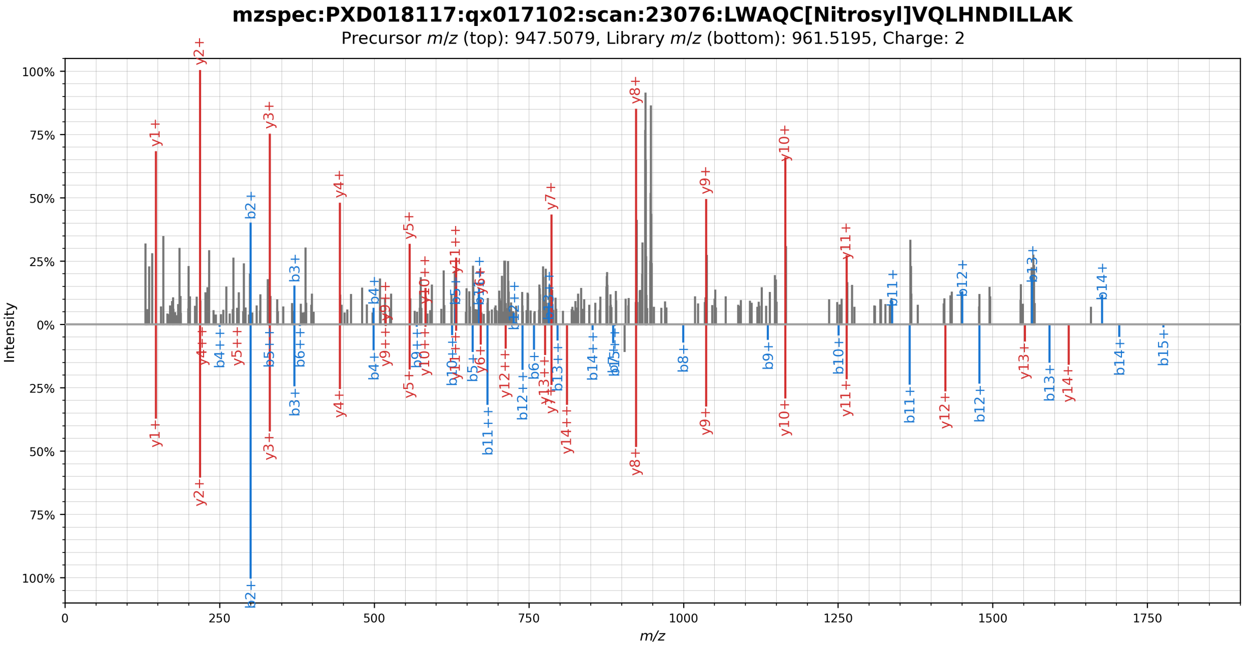

Fig. S43. Mirror plot showing the spectral library match (bottom) of a S-nitrosylated peptide originating from the SARS-CoV-2 Nsp7 protein (top). The S-nitrosylation was localized to residue C33 by visual inspection.

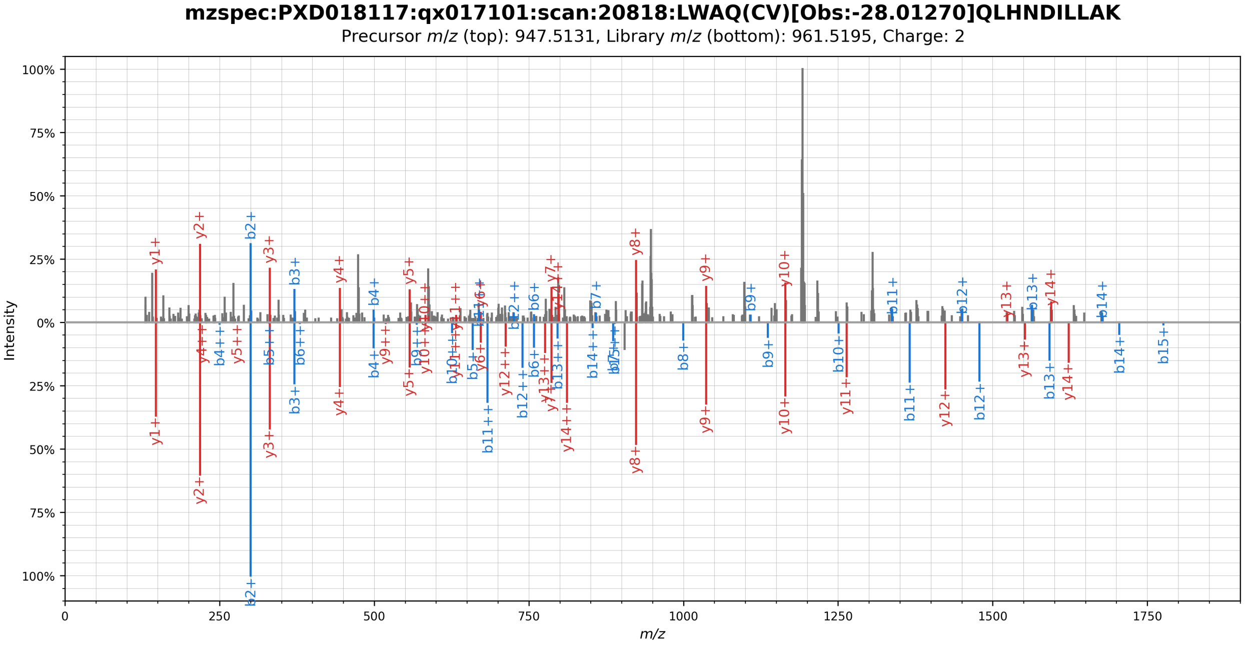

Fig. S44. Mirror plot showing the spectral library match (bottom) of a possibly S-nitrosylated peptide originating from the SARS-CoV-2 Nsp7 protein (top). The mass was localized to residue C33/V34 by visual inspection.

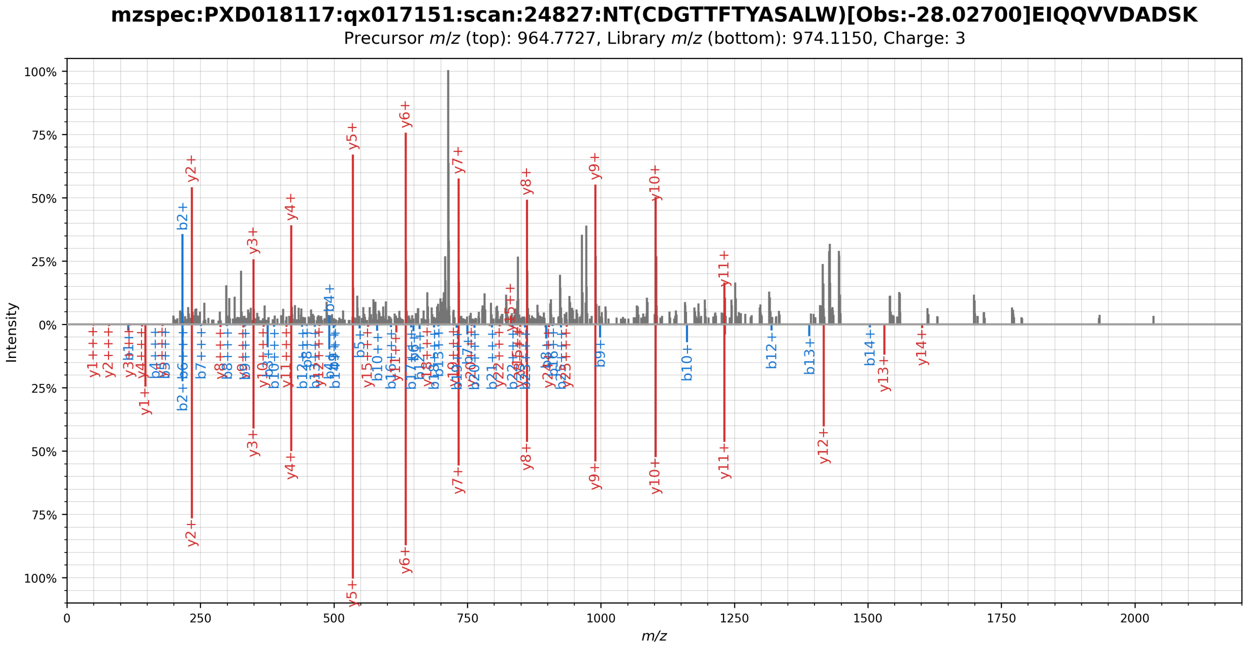

Fig. S45. Mirror plot showing the spectral library match (bottom) of a possibly S-nitrosylated peptide originating from the SARS-CoV-2 Nsp8 protein (top). The mass shift could not be localized by visual inspection.

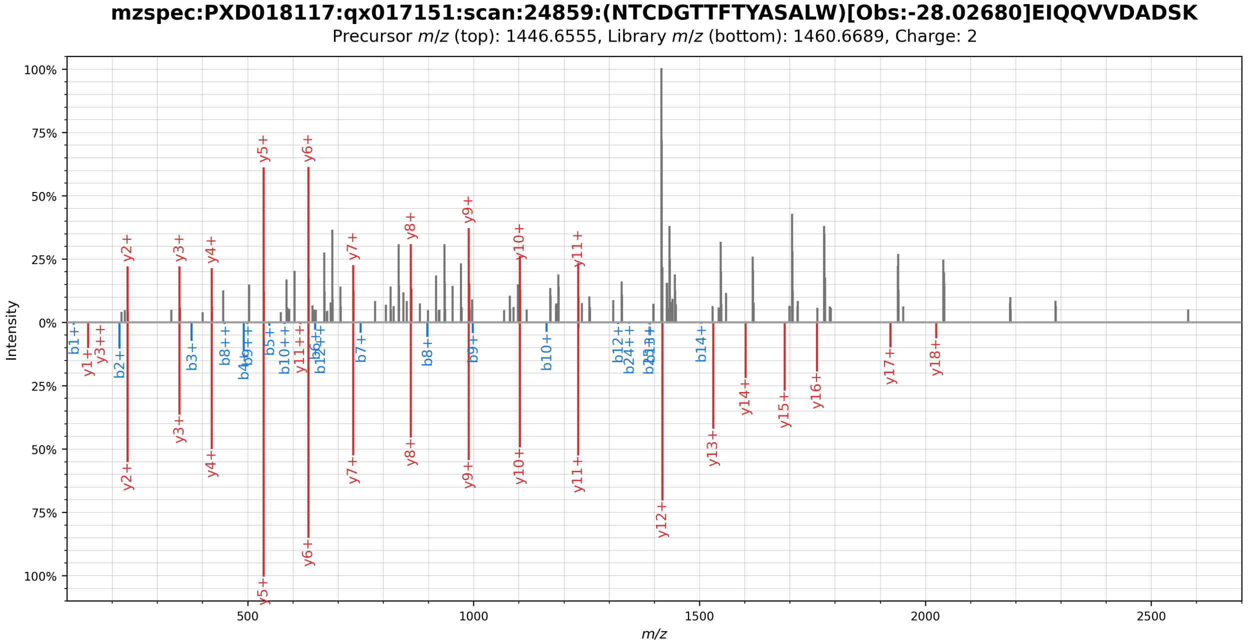

Fig. S46. Mirror plot showing the spectral library match (bottom) of a possibly S-nitrosylated peptide originating from the SARS-CoV-2 Nsp8 protein (top). The mass shift could not be localized by visual inspection.

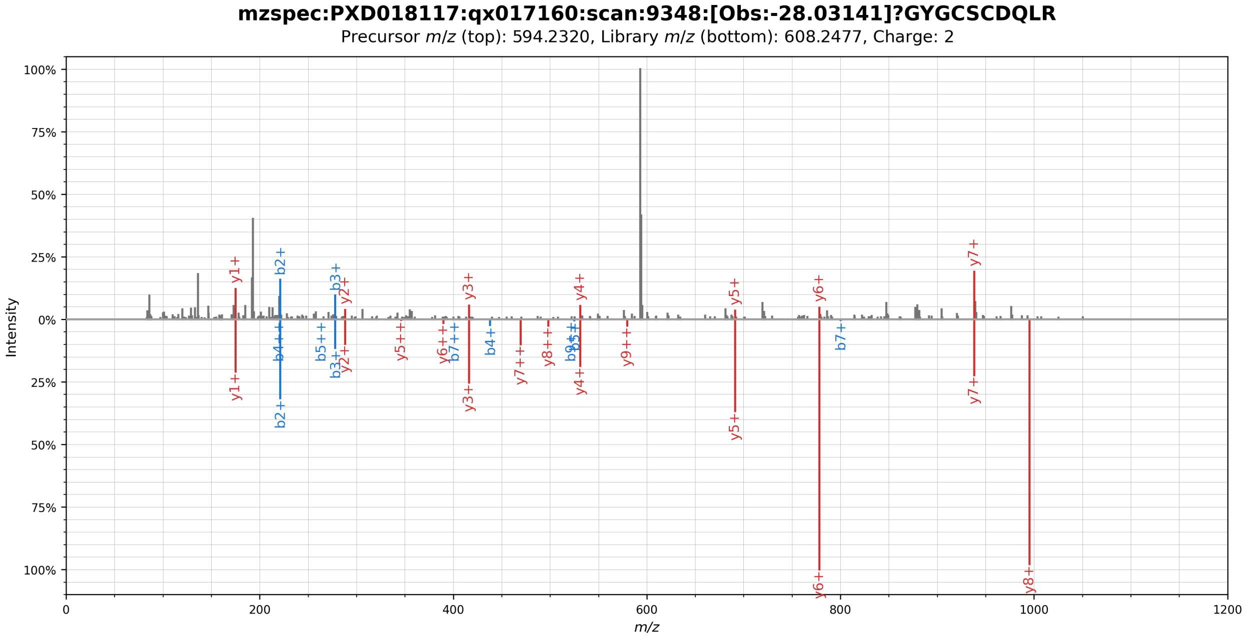

Fig. S47. Mirror plot showing the spectral library match (bottom) of a possibly S-nitrosylated peptide originating from the SARS-CoV-2 Nsp10 protein (top). The mass shift could not be localized by visual inspection.

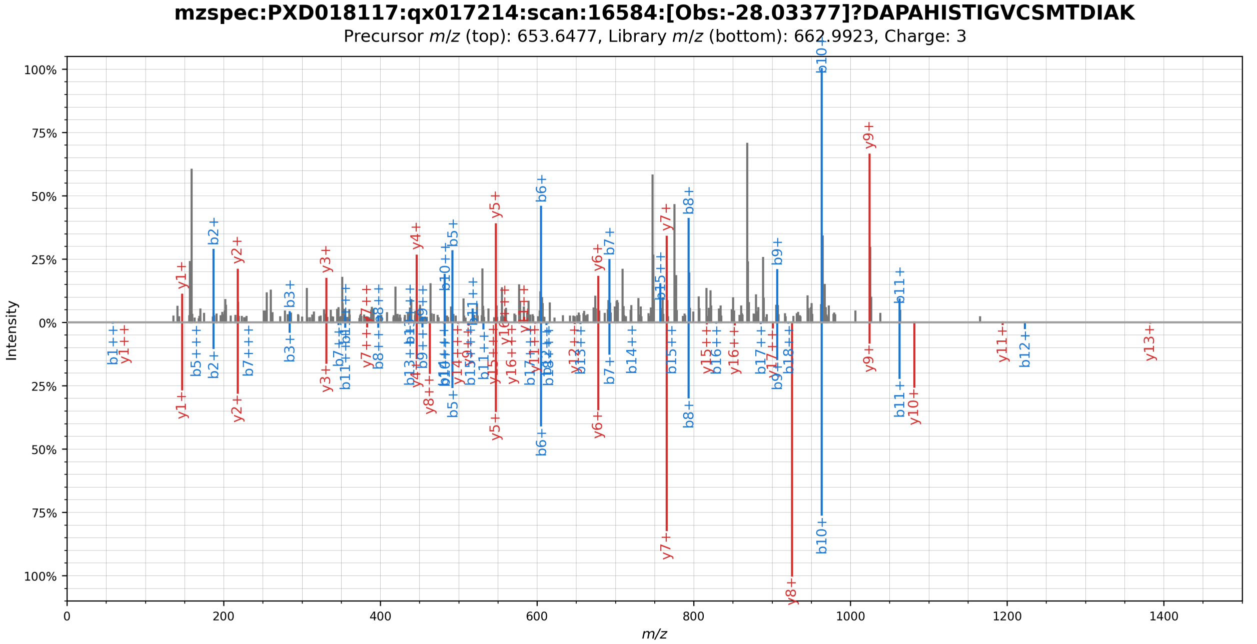

Fig. S48. Mirror plot showing the spectral library match (bottom) of a S-nitrosylated peptide originating from the SARS-CoV-2 Nsp15 protein (top). The S-nitrosylation was localized to residue C103 by visual inspection.

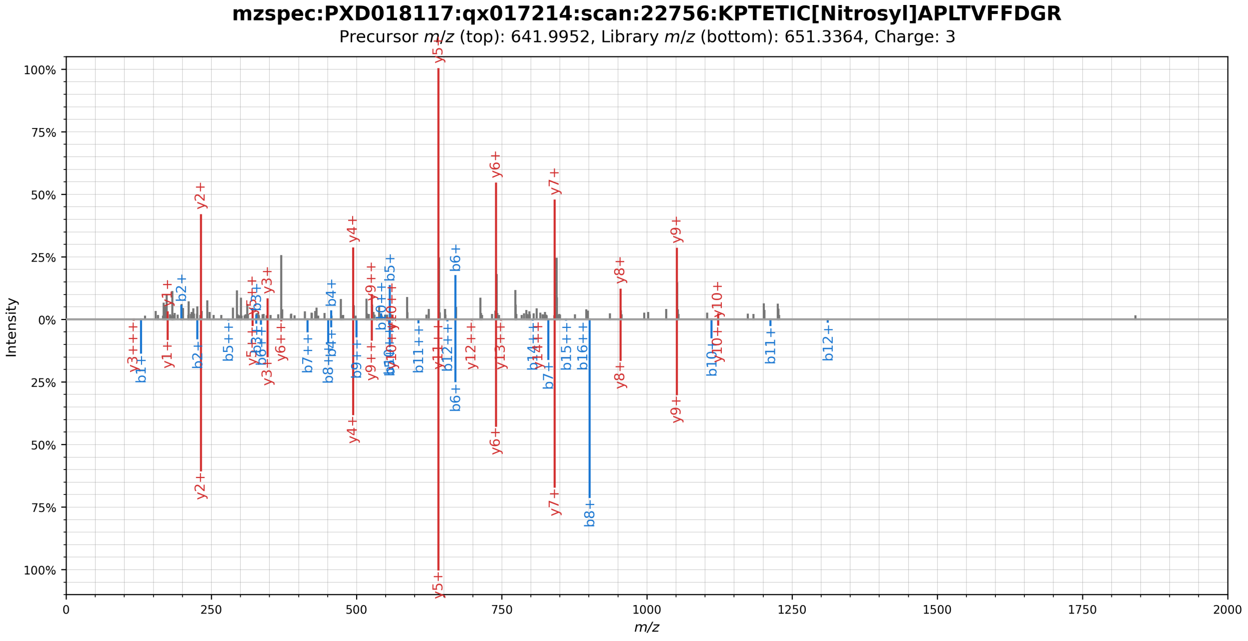

Fig. S49. Mirror plot showing the spectral library match (bottom) of a S-nitrosylated peptide originating from the SARS-CoV-2 Nsp15 protein (top). The S-nitrosylation was localized to residue C117 by visual inspection.

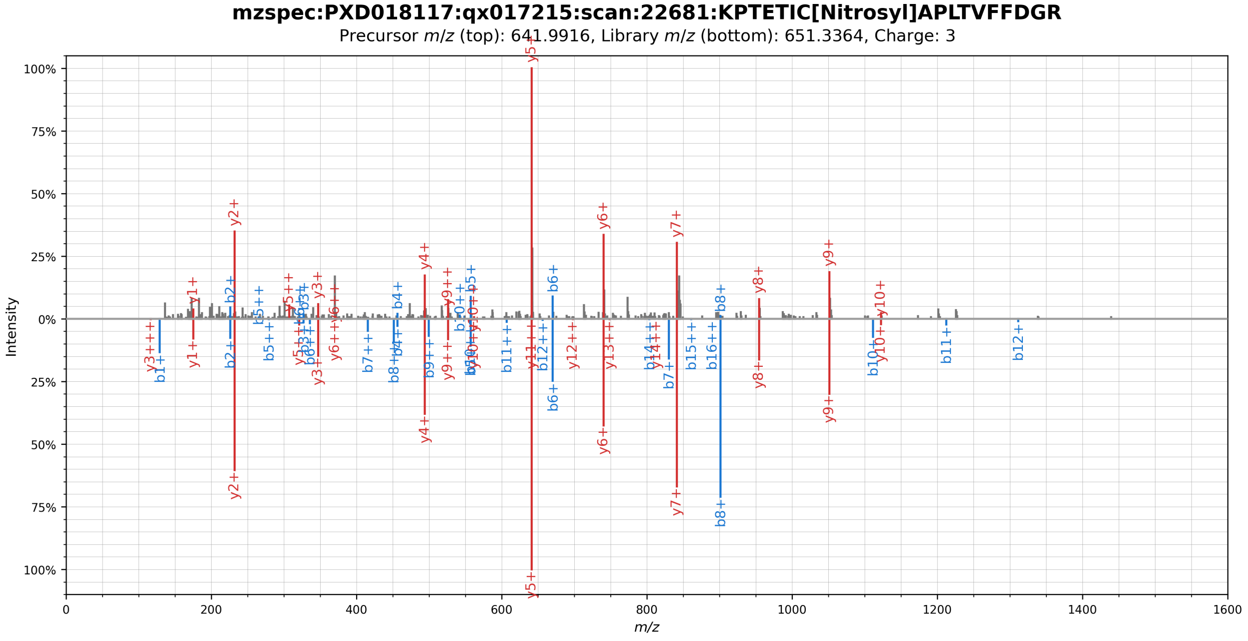

Fig. S50. Mirror plot showing the spectral library match (bottom) of a S-nitrosylated peptide originating from the SARS-CoV-2 Nsp15 protein (top). The S-nitrosylation was localized to residue C117 by visual inspection.

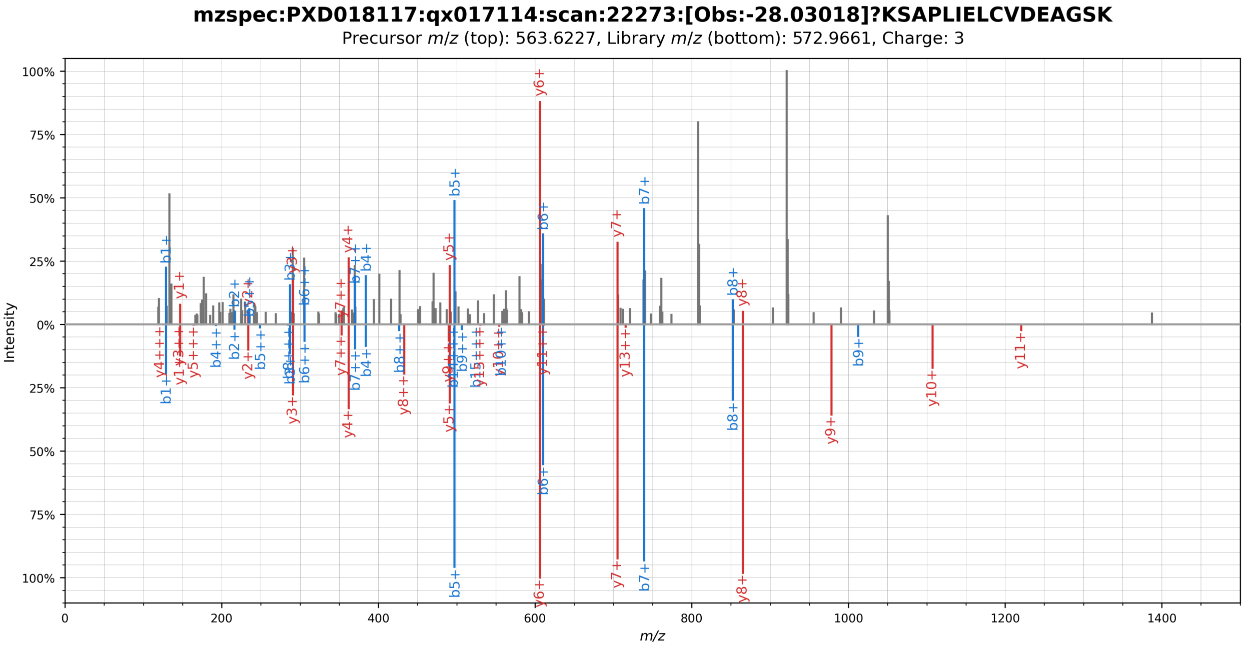

Fig. S51. Mirror plot showing the spectral library match (bottom) of a possibly S-nitrosylated peptide originating from the SARS-CoV-2 Orf8 protein (top). The mass shift could not be localized by visual inspection.

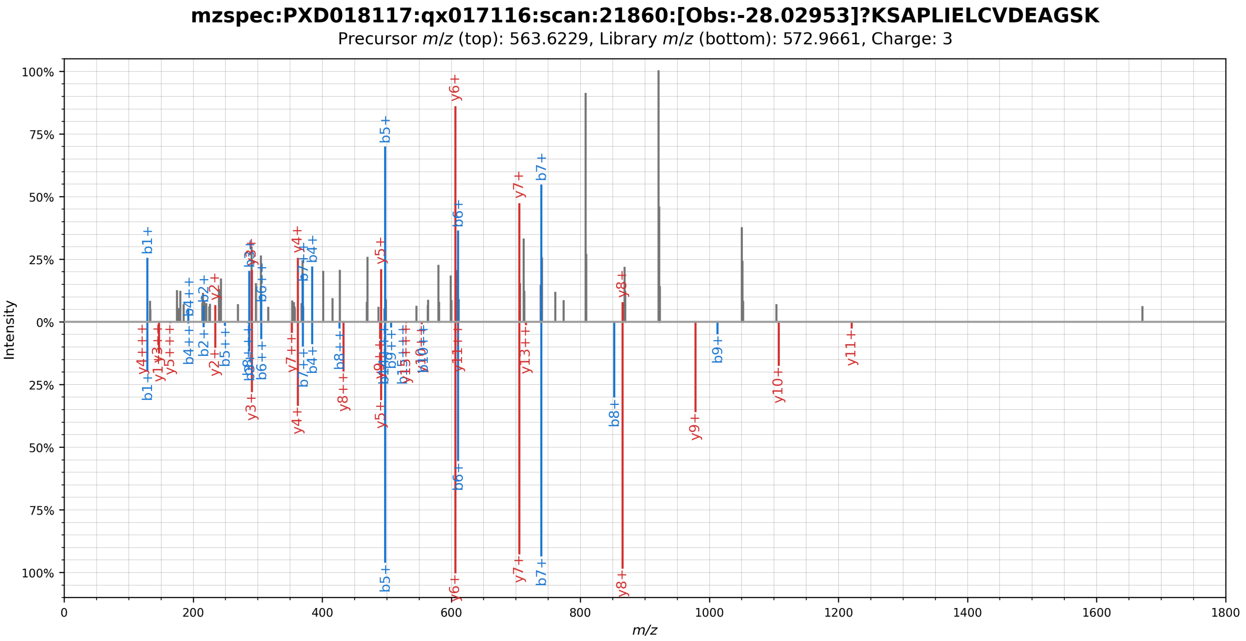

Fig. S52. Mirror plot showing the spectral library match (bottom) of a possibly S-nitrosylated peptide originating from the SARS-CoV-2 Orf8 protein (top). The mass shift could not be localized by visual inspection.
